# Muscle forces and the demands of turning while walking

**DOI:** 10.1242/bio.061883

**Published:** 2025-06-02

**Authors:** Steven G. Lautzenheiser, Patricia Ann Kramer

**Affiliations:** ^1^ Department of Anthropology, University of Tennessee, Knoxville, TN, USA; ^2^ Department of Anthropology, University of Washington, Seattle, WA, USA; ^3^ Department of Orthopaedics and Sports Medicine, University of Washington, Seattle, WA, USA

**Keywords:** Musculoskeletal model, Turning, Biomechanics, Lower limb

## Abstract

Turning is a ubiquitous feature of human locomotion and like straight path walking, requires muscular force both to propel the individual forward and to stabilize the trunk over the stance limb. The purpose of this study is to identify muscle force patterns while making a turn and compare them to those of straight path walking. Kinematic and kinetic data were collected from 10 adults who walked unshod at their self-selected normal velocity in four conditions: straight line, 45° and 90° turns with a sidestep, and a 45° crossover event. A musculoskeletal model was used to calculate muscle forces in the pelvis and lower limb. Statistical parameter mapping (SPM) was used to determine whether the muscle force patterns of the three turning conditions were different from walking in a straight path. We find that, overall, the muscles that stabilize the hip and ankle during walking demonstrate differences in timing and magnitude of their force patterns across all turning conditions.

## Teaser

**Summary:** Force patterns of the muscles that stabilize the hip and ankle show differences in timing and magnitude compared to force patterns of straight-path walking.

## INTRODUCTION

Bipedal locomotion is, arguably, the defining characteristic of the human lineage, and humans spend a substantial amount of time moving through their environment (Bassett et al., 2010, 2004; Pontzer et al., 2012). Consequently, the energetics and biomechanics of bipedal locomotion have been extensively investigated over decades (Bischof et al., 2010; Bowden, 1967; Cavagna et al., 1976; Cavagna and Kaneko, 1977; Cavanagh and Lafortune, 1980). Many parameters affect the energetics and biomechanics of normal gait, including the effects of speed (Bischof et al., 2010; Cavanagh and Lafortune, 1980; Morio et al., 2009; Steudel-Numbers and Tilkens, 2004; Stokes et al., 1979), terrain (Rue and Kramer, 2017; Voloshina et al., 2013), and burden status (Huang and Kuo, 2014; Rue and Kramer, 2017), to list a few parameters. Most of these analyses have used treadmills to simulate free movements in the environment (Jin, 2014; Morio et al., 2009), with far fewer conducted in more natural environments (Margaria, 1976; Minetti, 1995; Rue and Kramer, 2017).

When walking in a straight path, the process of one foot contacting the ground (the stance foot) while the other foot swings forward until it contacts the ground to begin the next step creates reciprocal pendular motion that conserves energy by transferring between its potential and kinetic forms (Cavagna et al., 1976). Two peaks in the ground reaction force (GRF) occur in the stance phase of straight path walking: after the foot makes initial contact with the ground but before midstance (Morris, 1977; Root et al., 1977) and when the foot pushes off against the ground (Morris, 1977). The GRF can be divided into three components relative to the direction of travel: forward (in the direction of travel), vertical, and a third component orthogonal to the first two, often designated as the side force. The vertical component reacts the gravitational and other dynamic forces of the body and usually has the largest magnitude of the three components. During the propulsive phase of walking in a straight path, vertical forces equal to at least 1.2 times body weight (Bowden, 1967) pass through the foot as it pushes off against the ground. Forward forces both brake, in early stance, and propel, in late stance, the individual and are approximately 0.25 times body weight during walking (Nilsson and Thorstensson, 1989). Side forces are the smallest component of the three, produced to balance and stabilize the body during straight path walking. Locomotion is, however, more than simply displacing the body through space in a straight path or on a treadmill, so these studies provide a limited understanding of human locomotion.

Humans modify their direction of travel often and for various reasons, including terrain perturbations (Minetti, 1995) and interactions with other individuals (Hader et al., 2015; Wilson et al., 2018). One important aspect of more natural movement that cannot be evaluated by straight path walking is turning. Turning happens constantly and seamlessly as an individual moves through their environment (Glaister et al., 2007). In a turn, the body is redirected from its original path and this change of direction disrupts the pendular motion of the lower limb. Any redirection of a mass requires a force, so turning the body requires a GRF during stance able to cause the directional change. At a minimum, a force that is applied at an angle to the original direction of travel, and parallel to the new direction, is required to redirect the individual. Ground reaction forces and ground reaction impulses (Glaister et al., 2008; Krafft et al., 2015; Orendurff et al., 2006; Saito et al., 2019; Strike and Taylor, 2009; Taylor et al., 2005), joint angles (Courtine and Schieppati, 2003; Hase and Stein, 1999), and electromyogram (EMG) patterns (Hase and Stein, 1999; Houck, 2003) have been shown to differ across the steps associated with directional changes. Additionally, turning involves significant energetic costs (Wilson et al., 2013) and there are associated postural adjustments made by the body that happen seamlessly (Hase and Stein, 1999; Hicheur et al., 2005; Imai et al., 2001; Sreenivasa et al., 2008; Xu et al., 2004).

How the individual muscles of the lower limb respond to, or create, these directional changes, however, remains unclear. While EMG studies have shown differences in muscle activation patterns (Houck, 2003), determining the contribution of each muscle is difficult. This can be especially complicated when muscles, such as gastrocnemius, cross two joints because muscles that span two joints are influenced by the joint moments of both joints (Neptune et al., 1999). To further our understanding of the forces produced during human movement, musculoskeletal modeling (MSM) has emerged as a technique that can be used to address these limitations (Delp et al., 2007; Kramer et al., 2022; Kramer and Sylvester, 2023; O'Neill et al., 2013; Sylvester et al., 2021). In MSM, forward dynamics determines motion based on applied forces and torques, whereas inverse dynamics computes the forces and torques required to achieve a specific motion (Buchanan et al., 2005). A key challenge in inverse dynamic analyses, known as the muscle redundancy problem, is determining how to distribute the net joint moments among individual muscles (Anderson and Pandy, 2001; Stanev and Moustakas, 2019). While some approaches to addressing this issue have distributed force based on the assumption of uniform muscle activation (Perry et al., 2011; Warrener et al., 2015) this is not well supported (Crowninshield and Brand, 1981). MSMs incorporate representations of individual muscles (or even portions of muscles) to address the muscle redundancy problem by minimizing a particular criterion, frequently the sum of muscle activations raised to a power (Anderson and Pandy, 2001). Furthermore, musculoskeletal modeling can account for the coactivation of antagonist muscles and biarticular muscles.

While the muscle forces of straight path walking are well documented, those of turning are less so. Turning makes up about 20-50% of the steps that are taken each day (Glaister et al., 2007) so understanding the muscular forces that produce changes of direction is imperative. The main objective of the present investigation is to identify muscle force patterns while making a turn and compare them to those of straight path walking. A musculoskeletal model was employed to calculate muscle forces in the lower limb. Statistical parameter mapping (SPM) was used to assess whether the muscle force patterns during the three turning conditions differed from those observed while walking in a straight line.

## RESULTS

The normalized muscle force profiles for the apex step, the step taken at the innermost point of a curved path or turn, for the three turns are shown in Figs 1–3, while those for the initiation step, the step that begins the transition from a straight path into a turn, are shown in Figs S1-S3. Raw muscle force profiles for the apex (Fig. S4-S6) and initiation (Fig. S7-S9) steps are shown in the Supplementary Material. Full details of the SPM analysis can be found in Figs S10-S21. An analysis of variance (ANOVA) with correction for repeated measures shows no statistically significant difference between walking velocities across all four turning conditions (*p*=0.08).

**Fig. 1.**
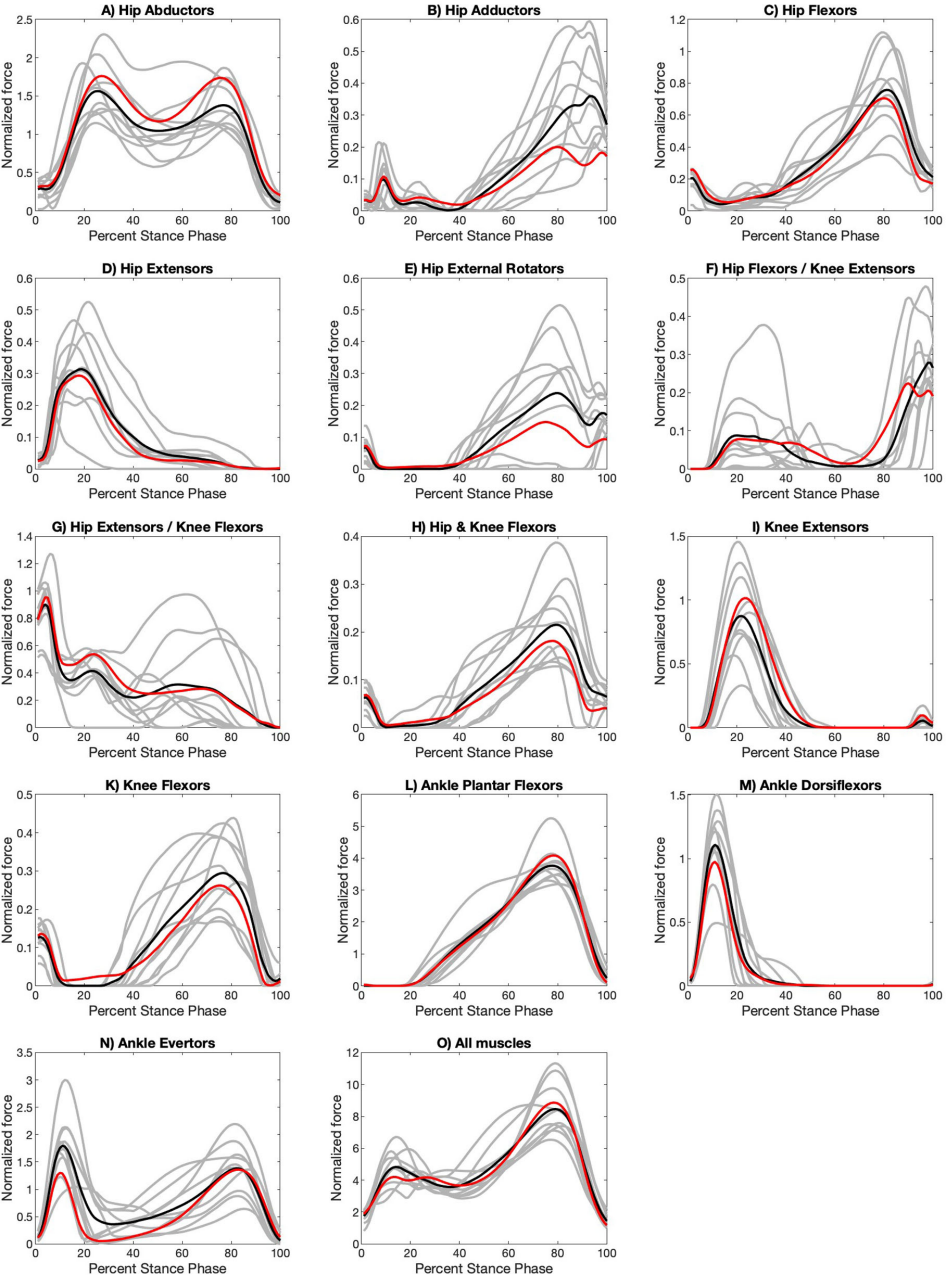
**Normalized functional muscle group force profiles for apex step during a 45° with a sidestep.** Grey lines are the average turning curve for each participant (two to five stance phases per participant). Black lines are the average of the 10 participant average turning curves. Red lines are the average of the 10 participant average curves for straight path walking (Sylvester et al., 2021). Turning force regions that are significantly different at *P*<0.003 from the values for straight path walking are indicated with grey shading. Full details of the SPM analysis can be found in Fig. S10.

**Fig. 2.**
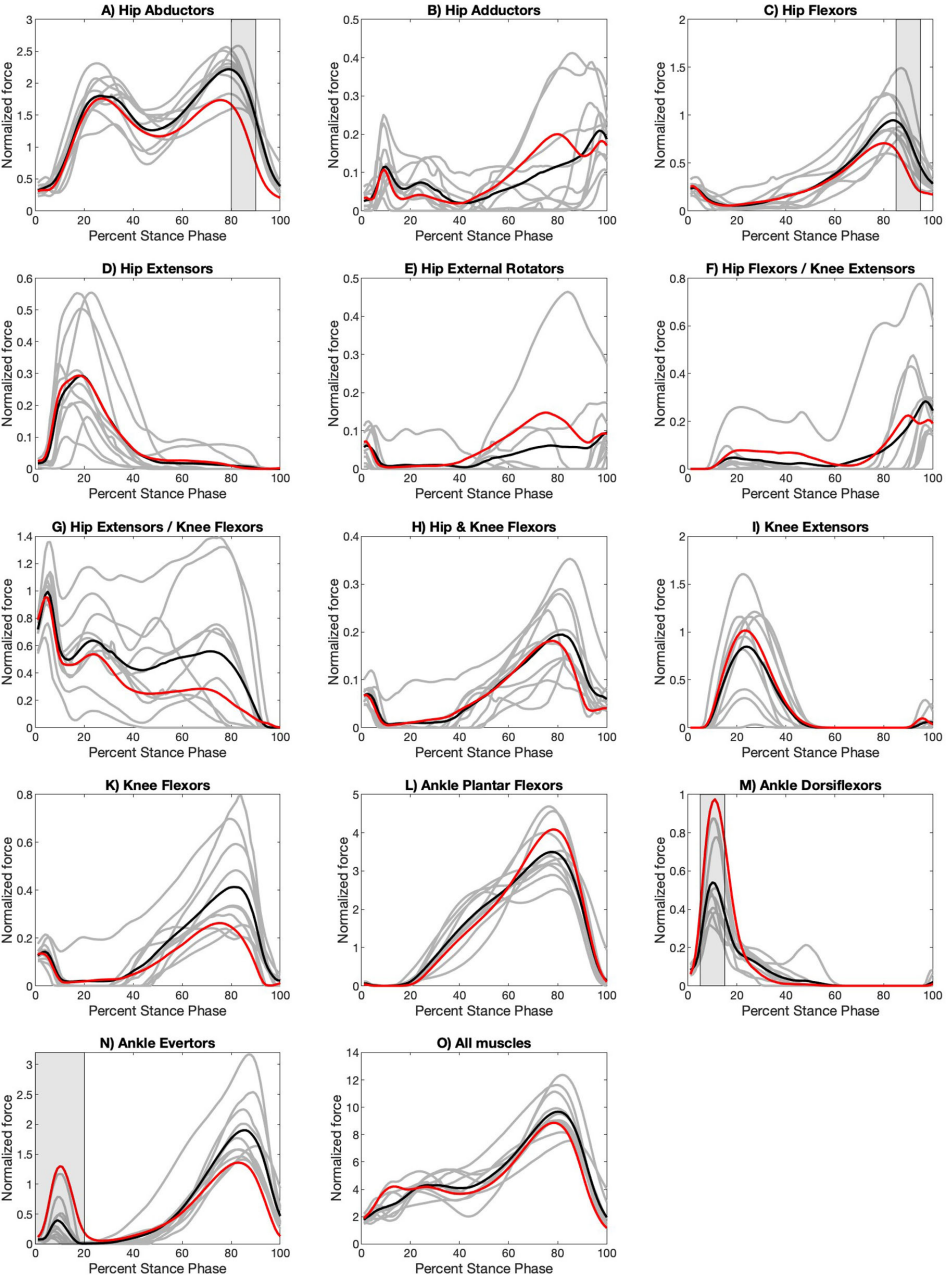
**Normalized functional muscle group force profiles for apex step during a 45° with a crossover.** Grey lines are the average turning curve for each participant (two to five stance phases per participant). Black lines are the average of the 10 participant average turning curves. Red lines are the average of the 10 participant average curves for straight path walking (Sylvester et al., 2021). Turning force regions that are significantly different at *P*<0.003 from the values for straight path walking are indicated with grey shading. Full details of the SPM analysis can be found in Fig. S11.

**Fig. 3.**
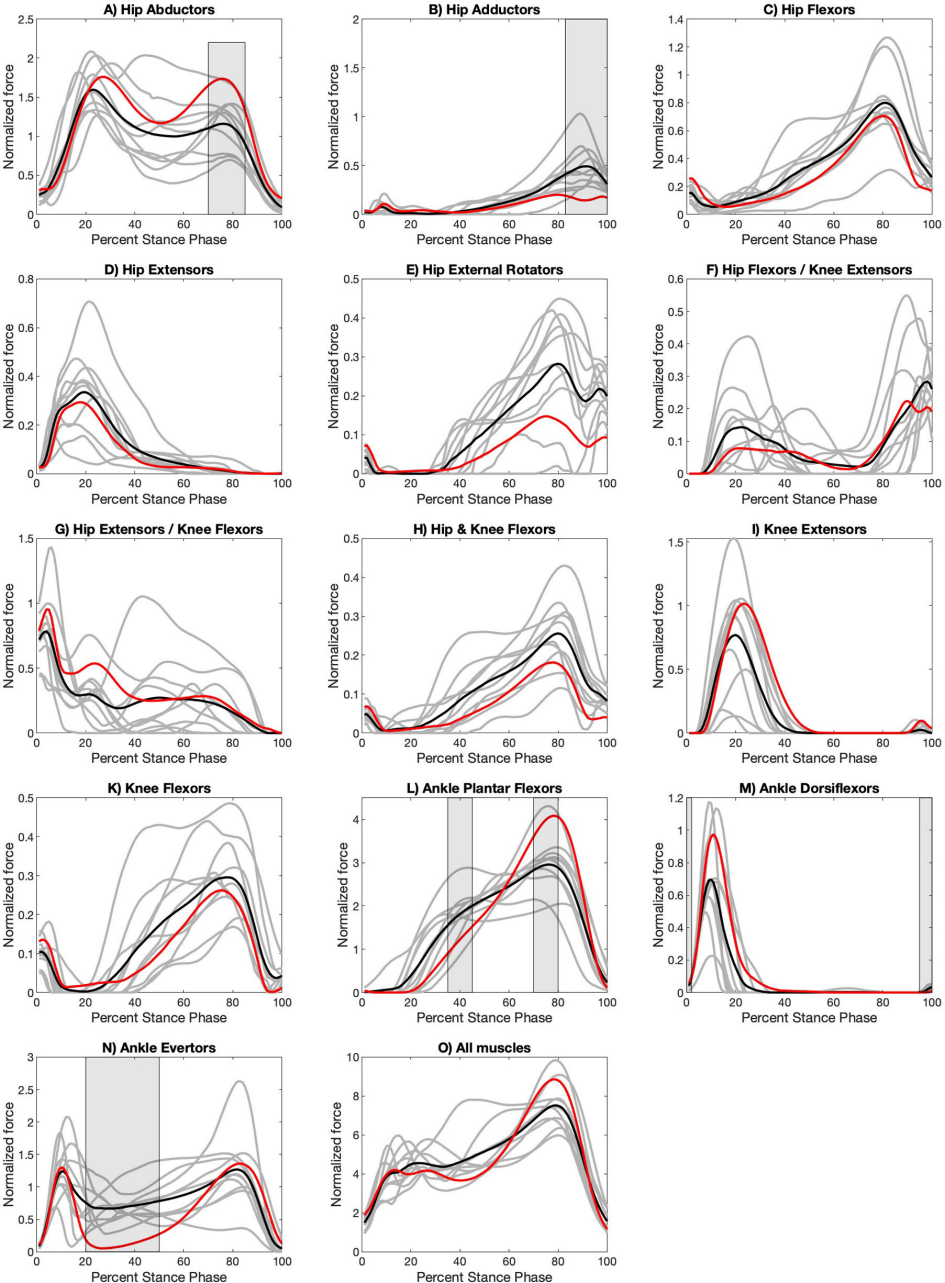
**Normalized functional muscle group force profiles for apex step during a 90° with a sidestep.** Grey lines are the average turning curve for each participant (two to five stance phases per participant). Black lines are the average of the 10 participant average turning curves. Red lines are the average of the 10 participant average curves for straight path walking (Sylvester et al., 2021). Turning force regions that are significantly different at *P*<0.003 from the values for straight path walking are indicated with grey shading. Full details of the SPM analysis can be found in Fig. S12.

For the apex step, some muscle forces acting across the hip and ankle joints differ from those of straight path walking for all types of turns (Figs 1–3; Figs S10-S12) but these differences manifest most clearly in the open 90° turn in both the magnitude and the duration of the forces. Ankle evertors produce higher forces for more of stance (Figs 1–3; Figs S10-S12), while ankle plantar flexors reach lower peak force but are active across more of stance (Figs 1–3; Figs S10-S12). Ankle dorsiflexor muscle forces are lower during a turn (Figs 1–3; Figs S10-S12). Hip coronal plane stabilization muscle forces are different with adductor muscle forces doubled in the 90° turn (Fig. 3) while abductor muscle forces are less (Fig. 3; Fig. S12). Muscle forces that act across the knee joint do not appear to differ between straight path and turns (Figs 1–3; Figs S10-S12).

## DISCUSSION

The GRF components required to make a turn differ from those required to move the body in a straight path (Glaister et al., 2008; Krafft et al., 2015; Orendurff et al., 2006; Saito et al., 2019; Strike and Taylor, 2009; Taylor et al., 2005). As an individual begins to turn, the body slows (i.e. brakes) in its current course to initiate the directional change. This change requires the application of a force at an angle to the initial direction of travel and in the new direction of travel. In a turn, the lateral and forward forces are effectively ‘swapped’ (Fig. S22), changing the magnitude and direction of the GRF (Glaister et al., 2008). Due to the changes in direction of the GRF, the activity in muscles also changes to maintain stability of the hip, knee, and ankle while walking.

We predicted that turns would produce muscle forces that differed at the hip, knee, and ankle joints. We found that some muscle groups that cross the hip and ankle are different, but those that cross just the knee are not (Figs 1–3). The force patterns of the ankle dorsiflexors show a difference in the apex step of all turning conditions. The ankle dorsiflexor activate in the early portion of the stance to control the descent of the foot and stabilize the leg at the ankle after initial contact. During the apex step of both a 45° turn with crossover and 90° turn with a sidestep the force patterns of the ankle plantar flexors, ankle evertors, and hip abductors differ, potentially to increase stabilization of the trunk at the hip and leg at the ankle. In a 45° turn with a crossover, stabilization of the hip and ankle is necessary to prevent an individual from falling over as the swing leg crosses the path of progression. Finally, in a 90° turn with sidestep, stabilization at the hip and ankle braces the stance leg as the new path of progression is perpendicular to the original path. Additionally, in the 90° turn with a sidestep the hip adductors provide additional stabilization to the stance leg to resist the application force in the original direction. Also, of note and a topic for future evaluation, some muscle functional groups appear to produce consistent changes among participants while others did not (Figs 1–3). This inconsistency is also true for straight path walking (Sylvester et al., 2021).

Turns change the magnitude and timing of the muscle forces, prompting the question: how does this change of magnitude and timing affect the skeletal elements of the lower limb? Mechanical forces, and the strains that they induce in musculoskeletal elements, play a critical role in bone function and development (Frost, 1982). The mature shape of each bone is influenced by the forces to which it is exposed, as bone is resorbed or added in response to load (Chen et al., 2010; Frost, 1982). The direction that a force is applied is as important as the force's magnitude in inducing bone strain and, hence, in influencing bone morphology. For example, a bone may be strong in compression (resulting from axial forces), but less capable in bending (resulting from shear forces) (Claes et al., 1998; Ruff et al., 2006).

The lateral load required to turn potentially produces an osteological response in different areas of the bone (i.e. one due to bending stress, which is characterized by compressive and tensile regions; Frost, 1982) than does an axial compressive load (which creates uniform compression). Most functional analyses of bone strain (e.g. Al Nazer et al., 2008; Farris et al., 2019; Fluit et al., 2014; Lovejoy et al., 1976; Sylvester and Kramer, 2018; Trepczynski et al., 2012) utilize muscle forces from straight path walking or standing in their evaluations. Consequently, bone strain profiles from these analyses may miss important muscle loading combinations. Given that injury can occur when bones and soft tissue experience applied loads in directions that exceed their capabilities (Jin, 2014; Scott and Winter, 1990), an evolutionary and ontological response is expected to accommodate frequent activities, as both straight path walking and changes of direction are. We see these results as an initial step to better understand how the musculoskeletal elements of the lower limb respond to turning. It is our intention to continue to continue to utilize these data, not only for understanding the evolution of human gait but also as a way to enhance treatment protocols and rehabilitation practices to mitigate the impacts of turning on the lower limb. One individual appears to respond to the turning conditions with lower walking velocities, we intend to further explore the differences in velocity across the walking conditions to better understand how individuals respond to directional changes.

### Limitations

Despite the significant results we obtained, several characteristics of the study limit it. The force plates that were used to collect these data are positioned in a straight path relatively close to each other and this arrangement was not customizable. While we were able to collect force data from the step in which the change of direction occurs, as well as the two steps before the turn, we were unable to collect the forces generated from the step directly after a turn occurred. Additionally, this study only looks at right turns. While we do not expect different results for a left turn, further work should look at left turns. Nonetheless, our results demonstrate important implications for lower limb biomechanics.

### Conclusions

Here, we show the timing and magnitude of the muscle forces of the lower limb during a directional change. Turning increases the force magnitudes and changes timings in force production in several of the muscle functional groups of the stance leg. Future work should address whether these changes are consistent among individuals and how the muscle forces of turns impact strain distributions in the bones of the lower limb.

## MATERIALS AND METHODS

### Subject population

Ten healthy participants (five females, five males) were recruited for this study, which is an extension of previously described work (Sylvester et al., 2021). All participants were free from self-reported lower limb injuries. The University of Washington's Institutional Review Board approved all procedures of this study (IRB#: STUDY00001125) and informed consent was obtained from all subjects. Participant anthropometrics including age (male: 31.8±13.8 years; female: 34±5.4 years), stature (male: 1.66±0.05 m; female: 1.77±0.05 m), body mass (male: 73.4±17.5 kg; female: 85.2±6.2 kg), and BMI (male: 26.5±5.5 kg/m²; female: 27.2±1.6 kg/m²) (Table S1), as well as a description of their associated straight path muscle force profiles, were previously reported (Sylvester et al., 2021).

### Experimental protocol

Kinetic and kinematic data were measured for individuals using four force plates (Kistler, Switzerland) and a 10 Opus 300 camera motion capture system (Qualisys, Sweden) in the Amplifying Movement & Performance laboratory of the University of Washington. The force plates are designated A-D in the direction of travel. Forty-nine infrared-reflective markers (5 mm hemispherical markers) were affixed to anatomical landmarks of the head, trunk, hip, thigh, knee, foot, and ankle. A full description of these markers is provided in the supplemental information (Appendix S1).

Using their self-selected normal pace, participants walked unshod the length of the gait lab (>15 m). Four walking conditions were explored for this study which included a straight walking path, 45° with a side-step to the right, 45° with a cross over, and a 90° with a side-step (Movies S1-S4). In a turn with a side-step, the left foot is the stance foot during the turn while the right foot (Movies S1-S4). In a turn with a cross over, the right foot is the stance foot, which then requires the left foot to cross over the right stance foot (Movie S3). Here, we only investigated a closed turn of 45° because turns of greater angles are challenging to accomplish without stopping. Before recording, participants were given the opportunity to practice the turning tasks to become acclimated. Each condition was repeated five times, resulting in 20 trials per participant. For all participants, the average velocity of each turning condition was calculated using the glabella marker and is reported in the Table S1. Trials during which multiple feet made contact with a particular force plate or a foot exceeded the force plate margins were discarded and immediately redone. In the straight path condition (Sylvester et al., 2021), the participants walked across all four force plates. In the turning conditions, participants walked in a straight path across force plates A and B and turned on force plate C. Marker data and the GRF were collected at 120 Hz and then filtered at 10 Hz with a fourth order, low pass zero-lag Butterworth filter. Calibration of the system yielded a limitation in its fidelity for marker data of 1 mm and force data of ±2.5 N for the forward, ±5 N side, and ±25 N vertical.

### Musculoskeletal model and simulation

As full description of the model and model simulation protocol can be found in Sylvester et al. (2021), an abridged description is provided here. We used the MoCap model that is part of the commercially available AnyBody Modelling System (v.7.3, AnyBody Technology, Denmark) to calculate muscle forces in the lower limb (Damsgaard et al., 2006). Each lower limb has six total degrees of freedom including three rotational degrees of freedom at the hip and one at each knee (flexion/extension), ankle (plantarflexion/dorsiflexion), and subtalar (inversion/eversion) joints. Forty-one anatomical muscles in each lower limb are represented by 169 muscle elements. For example, tibialis anterior is represented by six muscle elements (three in each limb).

Using the same protocol for the musculoskeletal simulation of walking trials (De Pieri et al., 2018), we scaled the model to each participant's mass and body segment dimensions and optimized marker locations (Andersen et al., 2009). An inverse dynamic analysis with a muscle redundancy algorithm was performed, which resulted in allocating muscle activations to achieve the measured accelerations and GRF.

### Model-derived variable and data processing

We exported force magnitudes from muscle elements in each lower limb during the stance phase of walking. In each turning condition, the stance phase was determined for each limb using GRF profiles and force plate contact detection output for force plate B (initiation step) and C (apex step). All stance phases were resampled to 1% increments of the stance phase using a custom written routine (MATLAB, USA). For each turning condition, a normalized, participant-specific muscle element profile was created by averaging the stance phase for each individual. Data files found to be corrupt or that contained significant marker dropout were not used when creating averages, resulting in two to five stance phases per individual. The overall force for each muscle was calculated by summing the force magnitudes produced by elements within anatomically defined muscles. Muscles that perform similar functions (e.g. knee extensors: vastus medialis, vastus intermedius, vastus lateralis) were then summed to produce a muscle functional group. Monoarticular muscles, muscles that cross and act on only one joint, and biarticular muscles, muscles that cross and act on two different joints, are placed in separate muscle functional groups (e.g knee extensors and hip flexors/knee extensors). The sum of all muscles was calculated (muscle sum). Finally, an average force curve profile for each muscle functional group and muscle sum was calculated by averaging the participant-specific curves. To facilitate comparisons, we normalized muscle forces by dividing each by participant body weight.

### Statistical analysis

An ANOVA with correction for repeated measures is used to determine if the velocity of each of the turning conditions is different from that of the straight path condition. This was performed using the individual trials for each walking condition; average velocity is reported in Table S1. To determine if the individual average muscle functional group forces in turns differed from those of straight path walking, we use statistical parameter mapping (SPM), a technique that allows for the evaluation of dependent variables that vary across a cycle and is particularly useful to assess gait variables that vary with time (Pataky et al., 2014, 2013). Analysis of variance with one factor (in this case, straight path versus turn) has been implemented in MATLAB through the spm1d package (spm1d.org). We established significance as an uncorrected *P*-value of 0.05 but we used a Bonferoni correction to establish a *P*-value of 0.003 (*P*=0.05/14 tests per turning condition) as indicating statistical difference.

## Supplementary Material

10.1242/biolopen.061883_sup1 Supplementary information
