# Supplementary material for: Muscle forces and the demands of turning while walking
Source: Biol Open. 2025 Jun 2;14(6):bio061883. doi: 10.1242/bio.061883 (PMC12182865; doi:10.1242/bio.061883)
Supplement: Supplementary information [file biolopen-14-061883-s1.pdf]

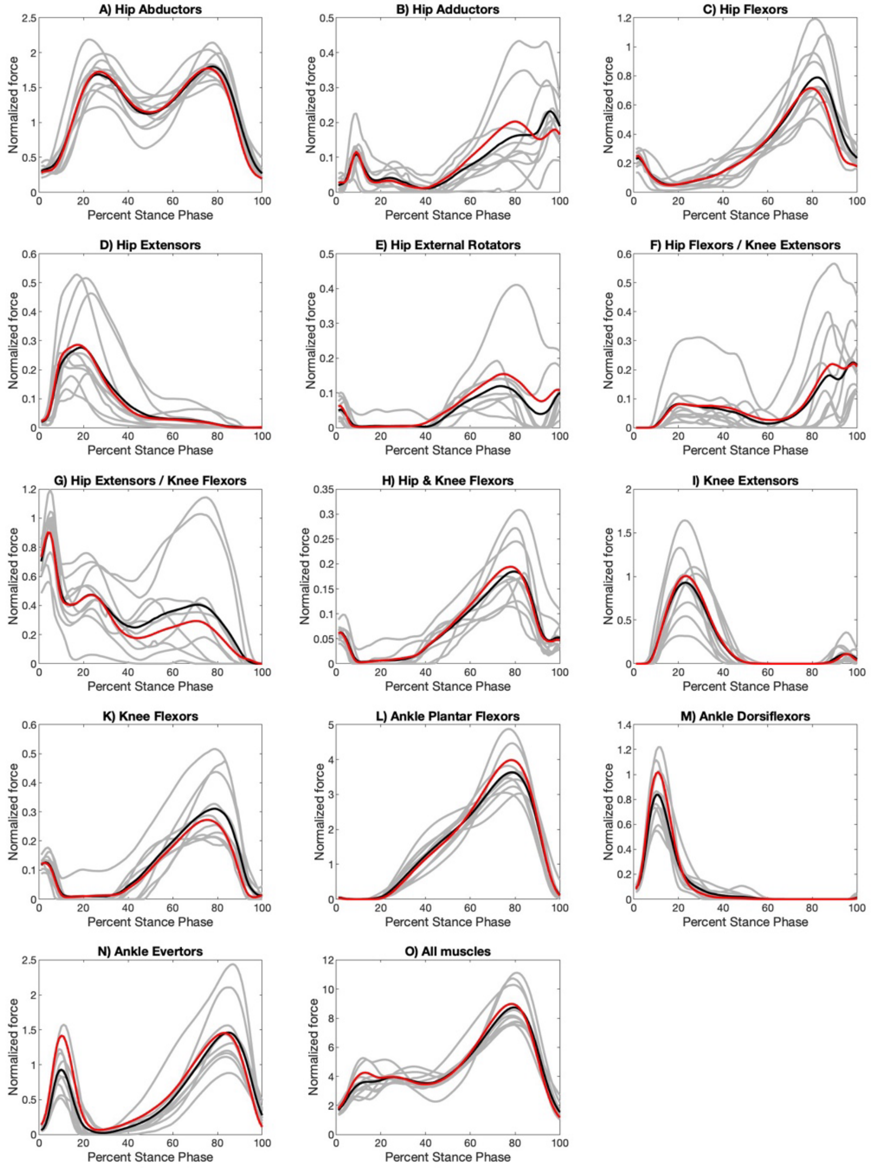

Fig. S1. Normalized functional group muscle force profiles for initiation step during a 45° with a sidestep. Grey lines are the average turning curve for each participant (two to five stance phases per participant). Black lines are the average of the ten participant average turning curves. Red lines are the average of the ten participant average curves for straight path walking (Sylvester et al 2021). Turning force regions that are significantly different at $p<0.003$ from the values for straight path walking are indicated with grey shading. Full details of the SPM analysis can be found in Fig. S13.

Biology Open • Supplementary information

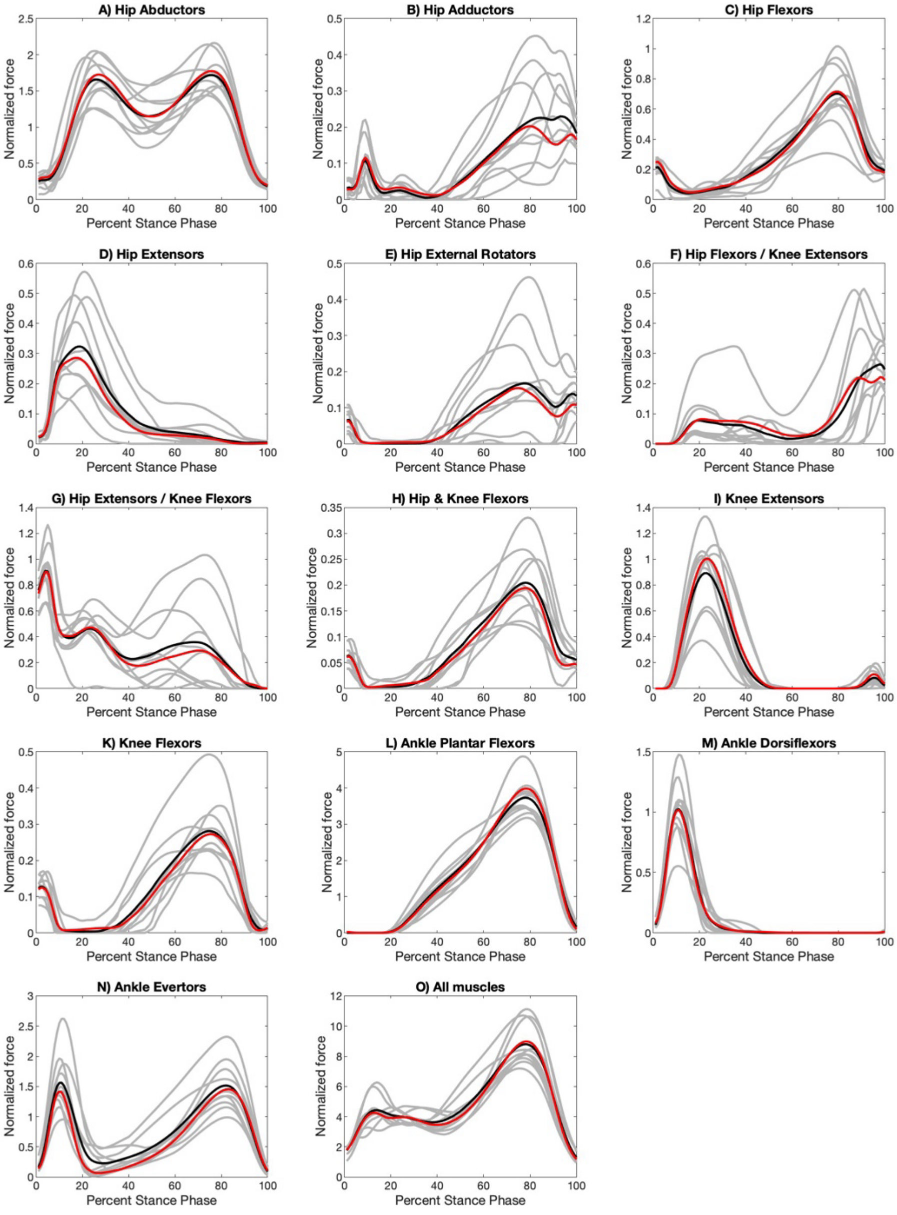

Fig. S2. Normalized functional group muscle force profiles for initiation step during a 45° with a crossover. Grey lines are the average turning curve for each participant (two to five stance phases per participant). Black lines are the average of the ten participant average turning curves. Red lines are the average of the ten participant average curves for straight path walking (Sylvester et al 2021). Turning force regions that are significantly different at p<0.003 from the values for straight path walking are indicated with grey shading. Full details of the SPM analysis can be found in Fig. S14.

Biology Open • Supplementary information

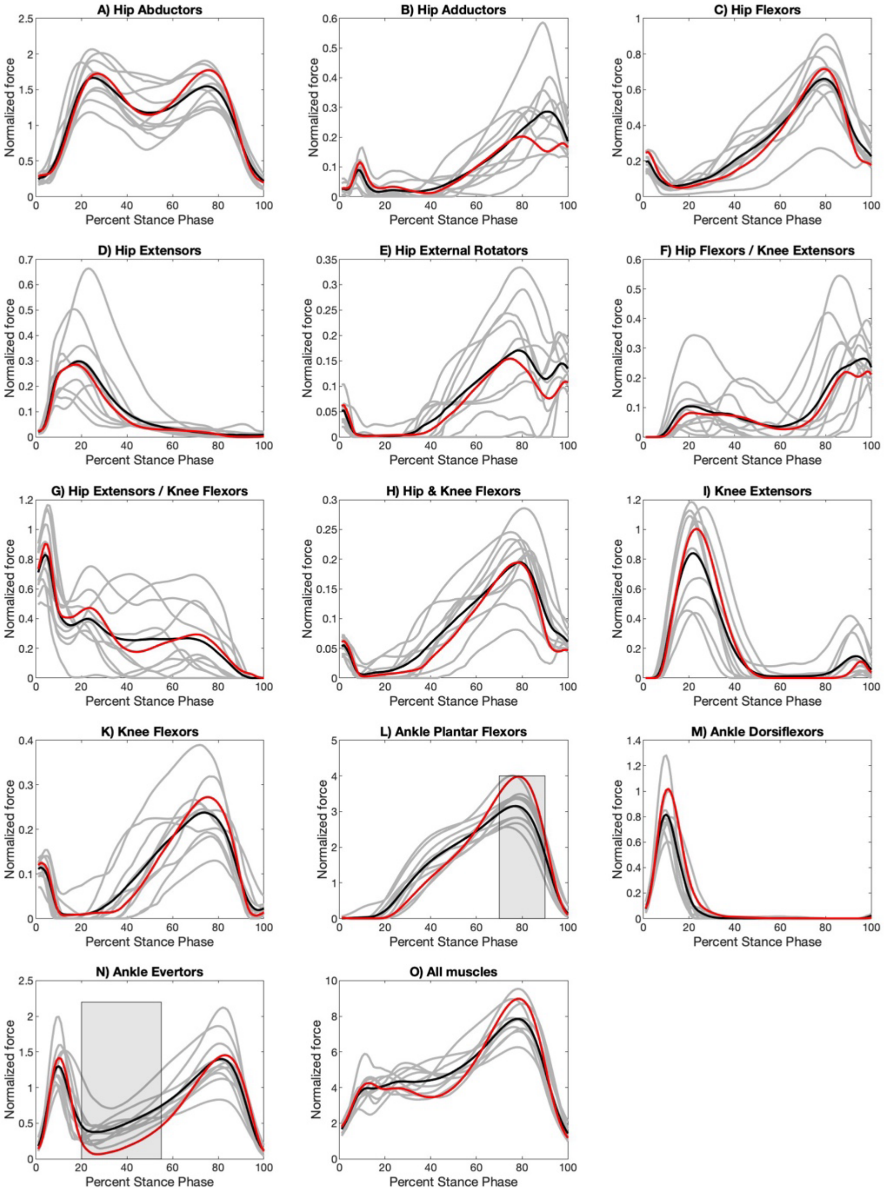

Fig. S3. Normalized functional group muscle force profiles for initiation step during a 90° with a sidestep. Grey lines are the average turning curve for each participant (two to five stance phases per participant). Black lines are the average of the ten participant average turning curves. Red lines are the average of the ten participant average curves for straight path walking (Sylvester et al 2021). Turning force regions that are significantly different at $p<0.003$ from the values for straight path walking are indicated with grey shading. Full details of the SPM analysis can be found in Fig. S15.

Biology Open • Supplementary information

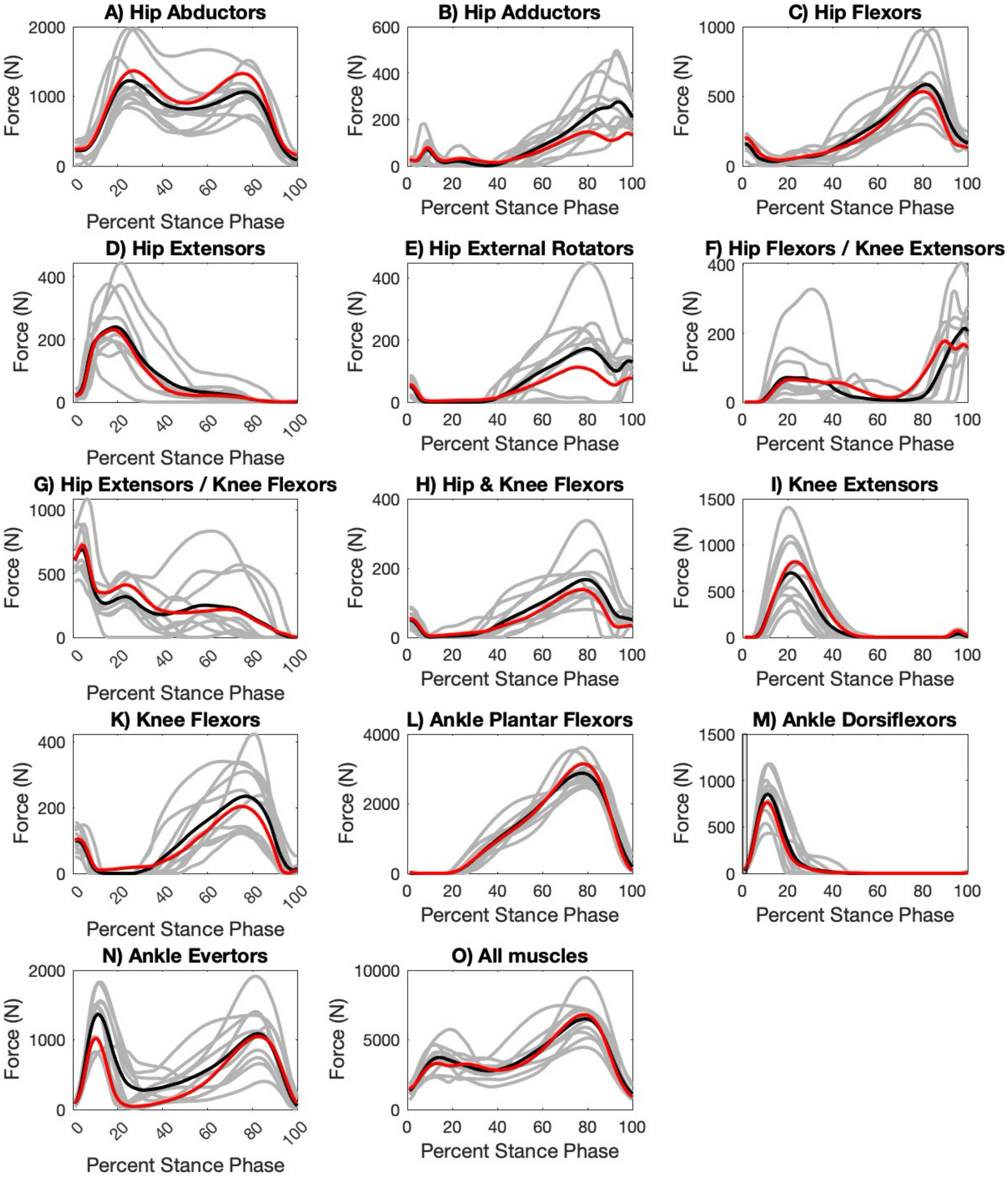

Fig. S4. Raw functional group muscle force profiles for apex step during a 45° with a sidestep. Grey lines are the average turning curve for each participant (two to five stance phases per participant). Black lines are the average of the ten participant average turning curves. Red lines are the average of the ten participant average curves for straight path walking (Sylvester et al 2021). Turning force regions that are significantly different at p<0.003 from the values for straight path walking are indicated with grey shading. Full details of the SPM analysis can be found in Fig. S16.

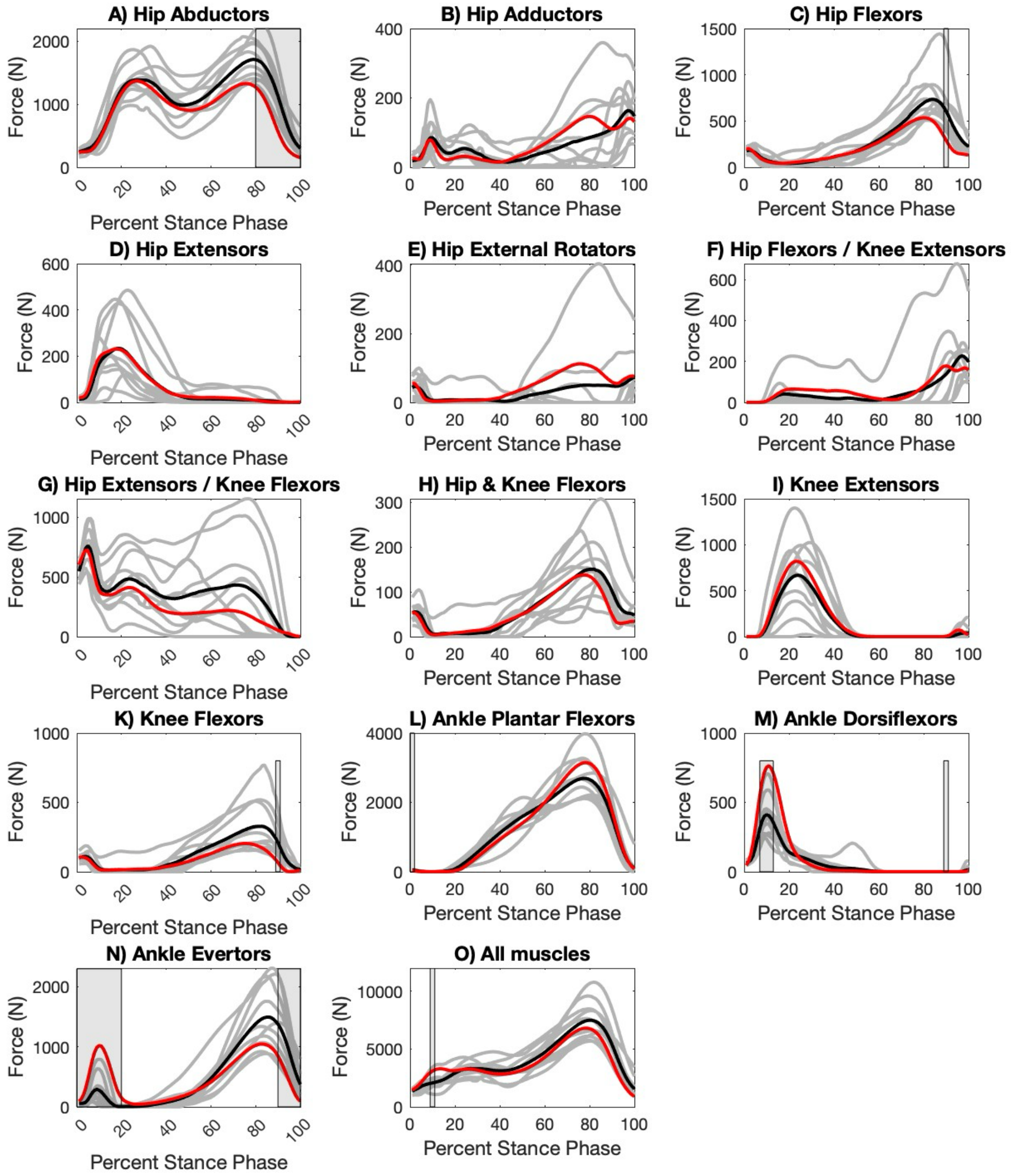

Fig. S5. Raw functional group muscle force profiles for apex step during a 45° with a crossover. Grey lines are the average turning curve for each participant (two to five stance phases per participant). Black lines are the average of the ten participant average turning curves. Red lines are the average of the ten participant average curves for straight path walking (Sylvester et al 2021). Turning force regions that are significantly different at $p<0.003$ from the values for straight path walking are indicated with grey shading. Full details of the SPM analysis can be found in Fig. S17.

Biology Open • Supplementary information

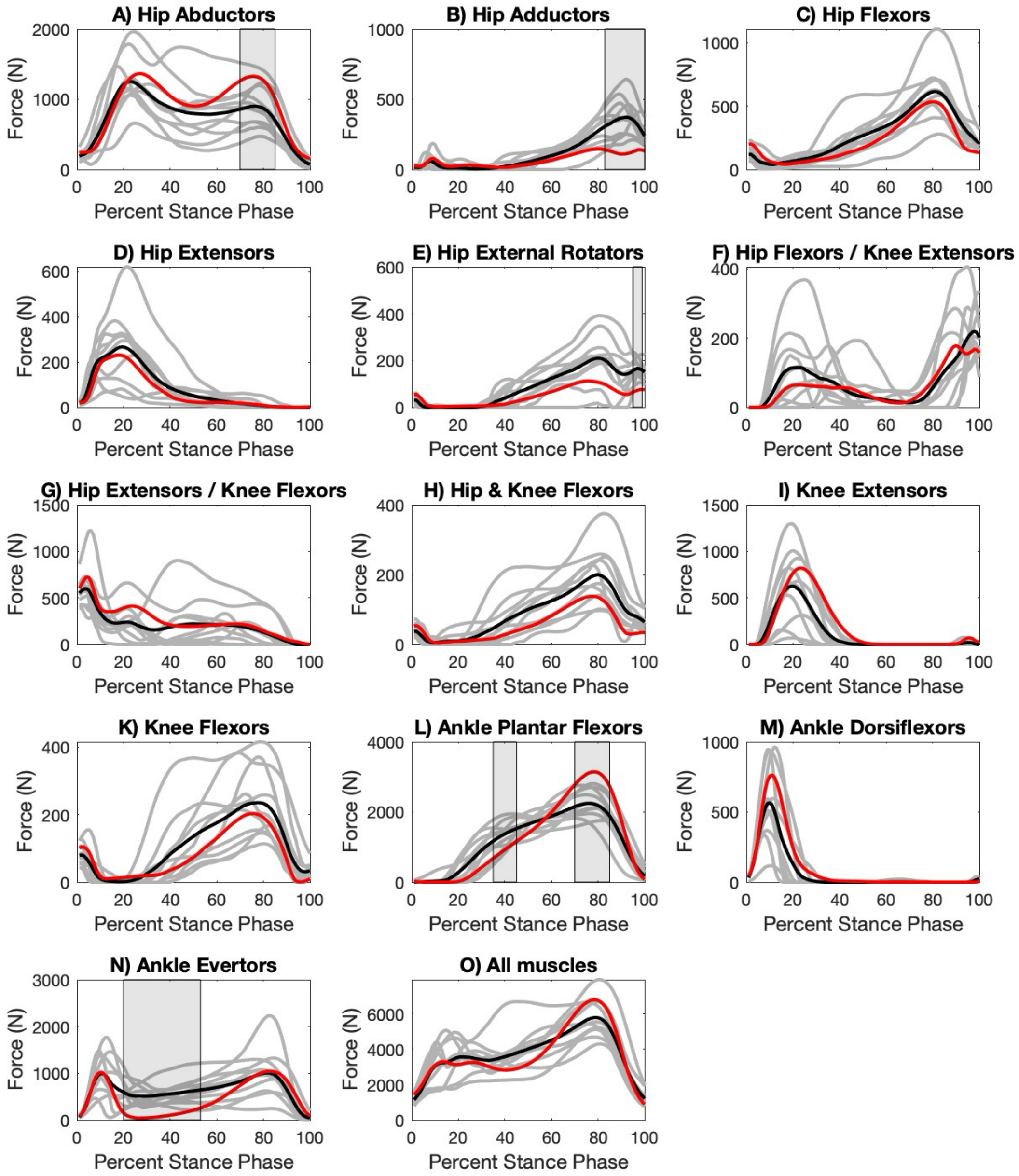

Fig. S6. Raw functional group muscle force profiles for apex step during a 90° with a sidestep. Grey lines are the average turning curve for each participant (two to five stance phases per participant). Black lines are the average of the ten participant average turning curves. Red lines are the average of the ten participant average curves for straight path walking (Sylvester et al 2021). Turning force regions that are significantly different at $p<0.003$ from the values for straight path walking are indicated with grey shading. Full details of the SPM analysis can be found in Fig. S18.

Biology Open • Supplementary information

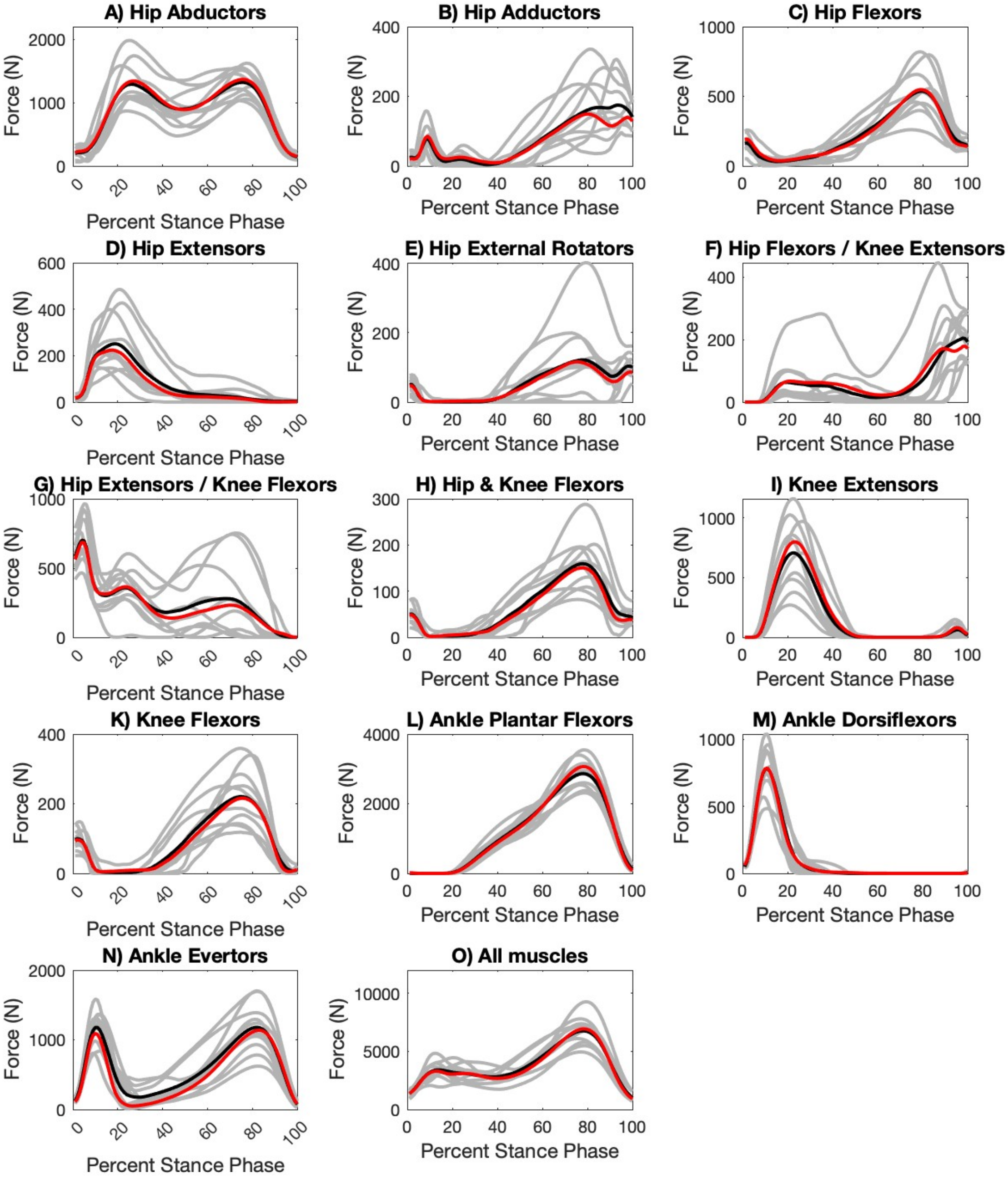

Fig. S7. Raw functional group muscle force profiles for initiation step during a 45° with a sidestep. Grey lines are the average turning curve for each participant (two to five stance phases per participant). Black lines are the average of the ten participant average turning curves. Red lines are the average of the ten participant average curves for straight path walking (Sylvester et al 2021). Turning force regions that are significantly different at p<0.003 from the values for straight path walking are indicated with grey shading. Full details of the SPM analysis can be found in Fig. S19.

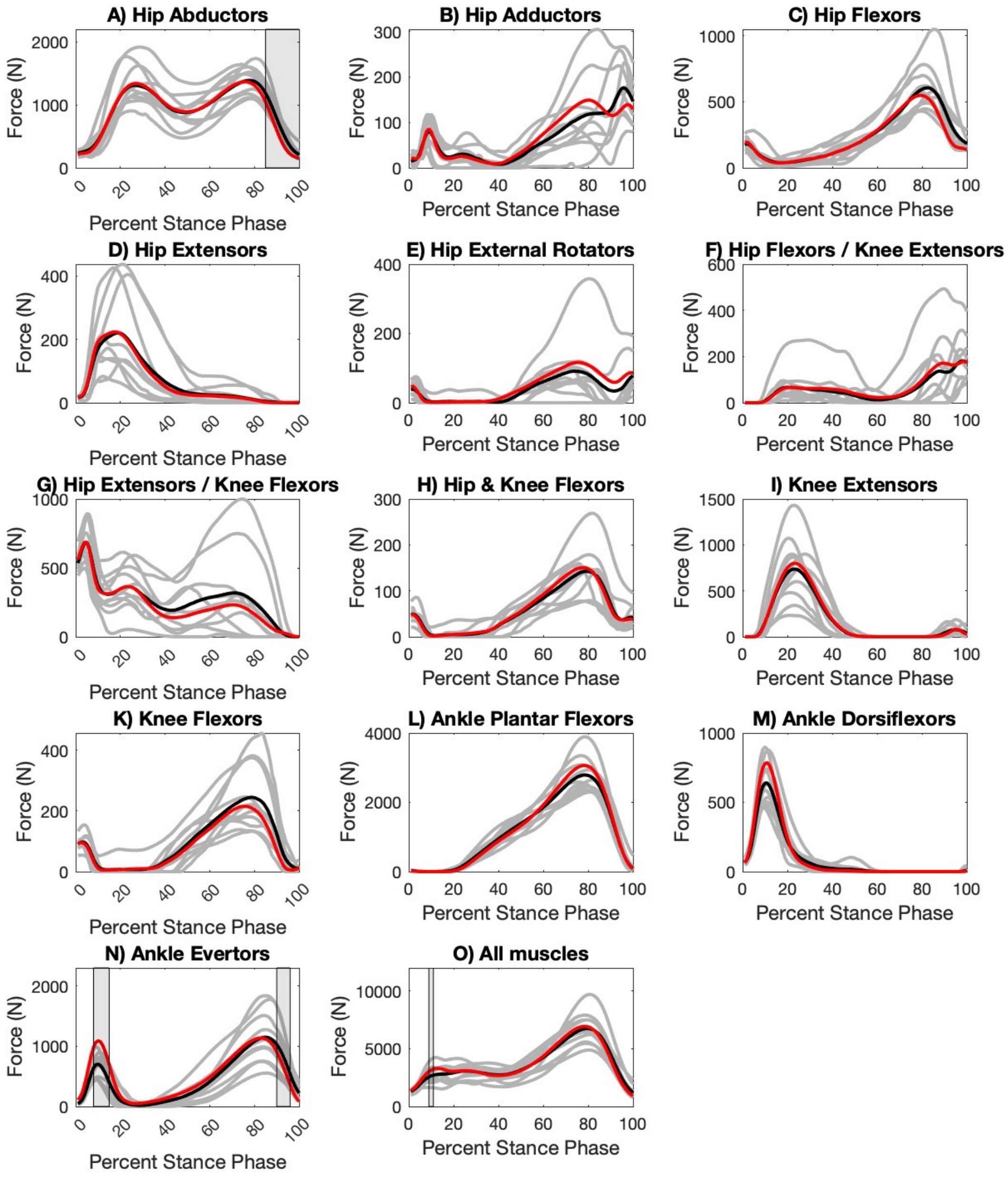

Fig. S8. Raw functional group muscle force profiles for initiation step during a 45° with a crossover. Grey lines are the average turning curve for each participant (two to five stance phases per participant). Black lines are the average of the ten participant average turning curves. Red lines are the average of the ten participant average curves for straight path walking (Sylvester et al 2021). Turning force regions that are significantly different at $p<0.003$ from the values for straight path walking are indicated with grey shading. Full details of the SPM analysis can be found in Fig. 20.

Biology Open • Supplementary information

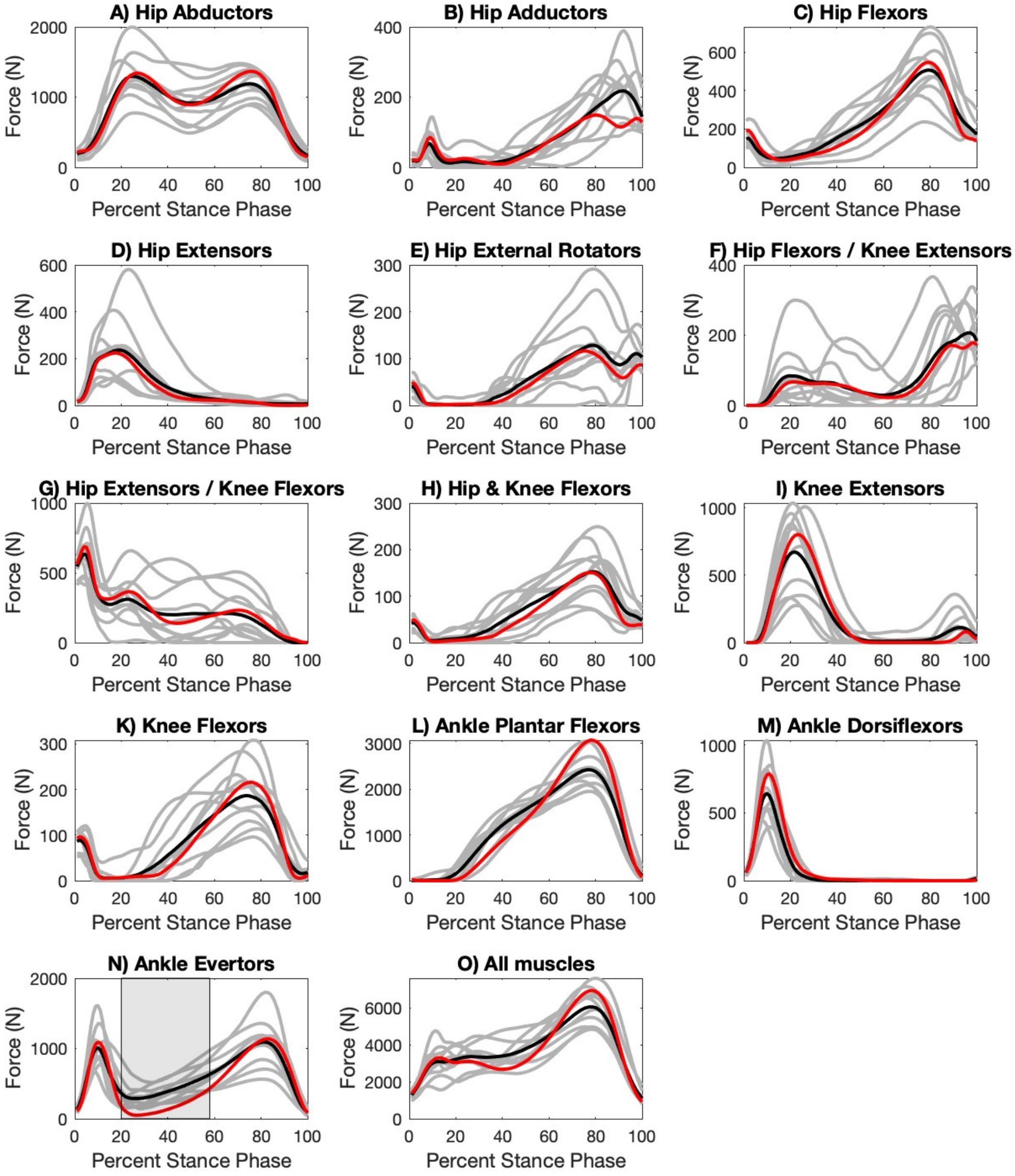

Fig. S9. Raw functional group muscle force profiles for initiation step during a 90° with a sidestep. Grey lines are the average turning curve for each participant (two to five stance phases per participant). Black lines are the average of the ten participant average turning curves. Red lines are the average of the ten participant average curves for straight path walking (Sylvester et al 2021). Turning force regions that are significantly different at p<0.003 from the values for straight path walking are indicated with grey shading. Full details of the SPM analysis can be found in Fig. S21.

Biology Open • Supplementary information

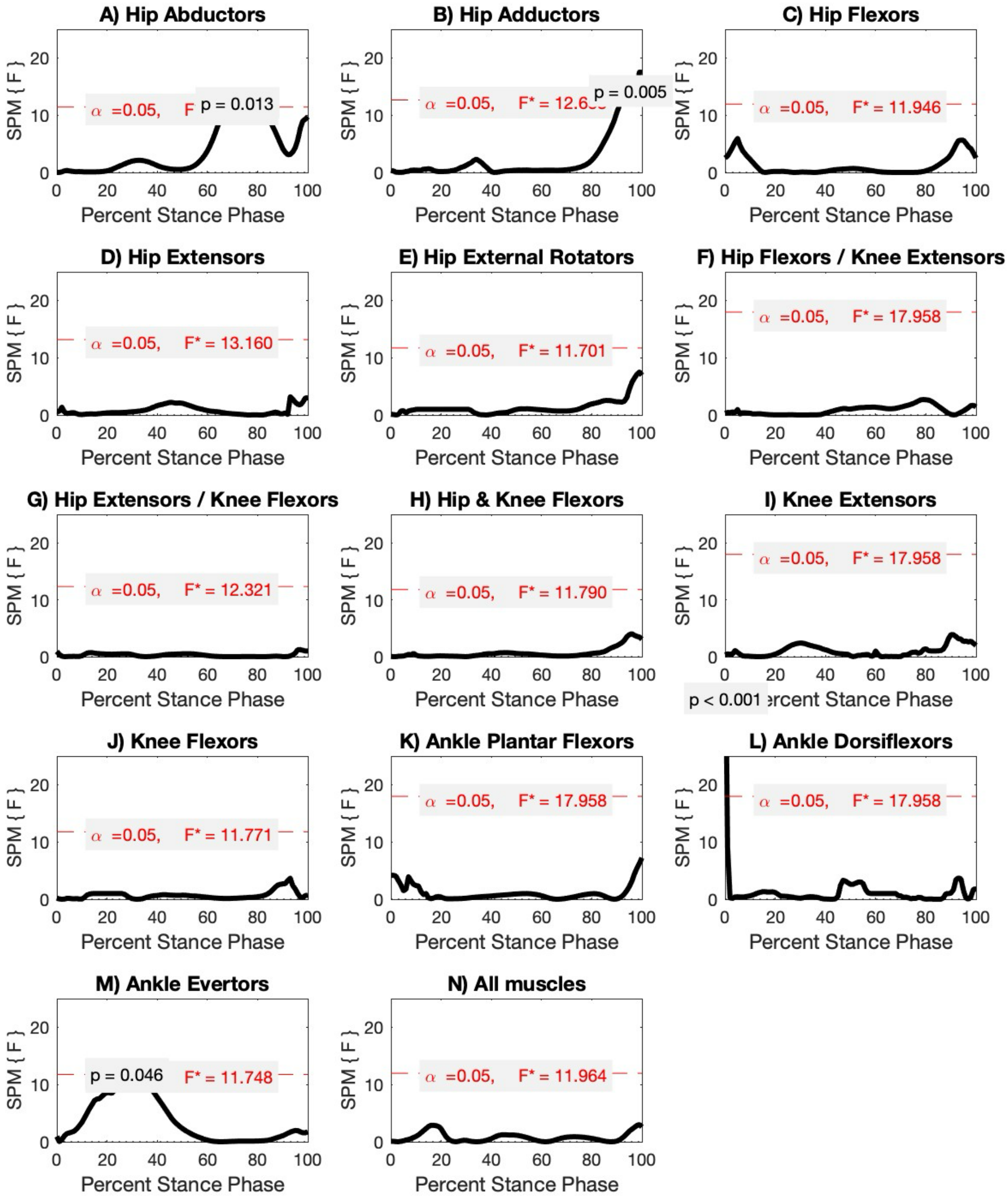

Fig. S10. Differences in normalized functional group muscle force profiles for apex step during a 45° with a sidestep revealed by the SPM. The statistically significant differences (for $p < 0.05$) are marked on the horizontal bar.

Biology Open • Supplementary information

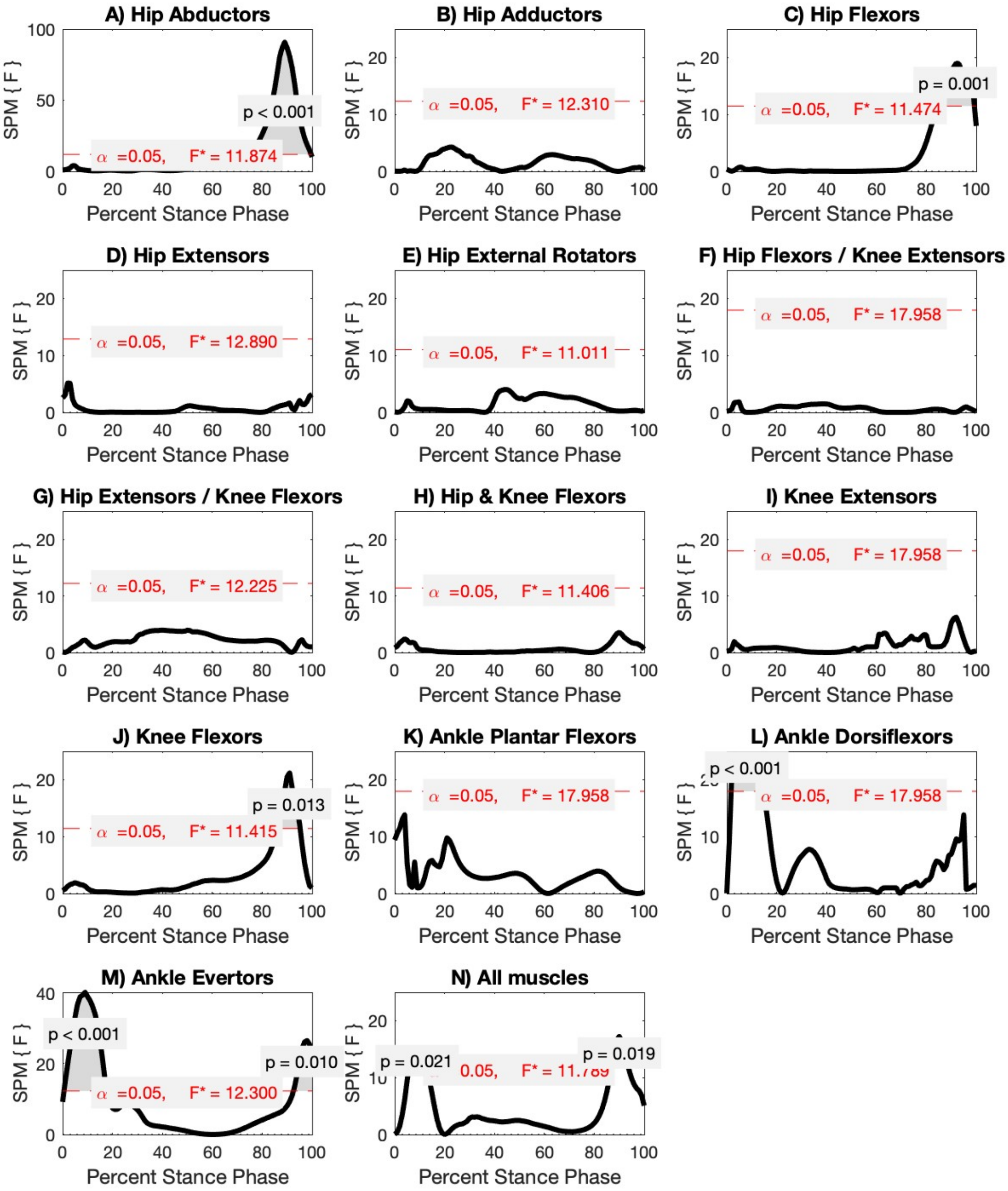

Fig. S11. Differences in normalized functional group muscle force profiles for apex step during a 45° with a crossover revealed by the SPM. The statistically significant differences (for $p < 0.05$) are marked on the horizontal bar.

Biology Open • Supplementary information

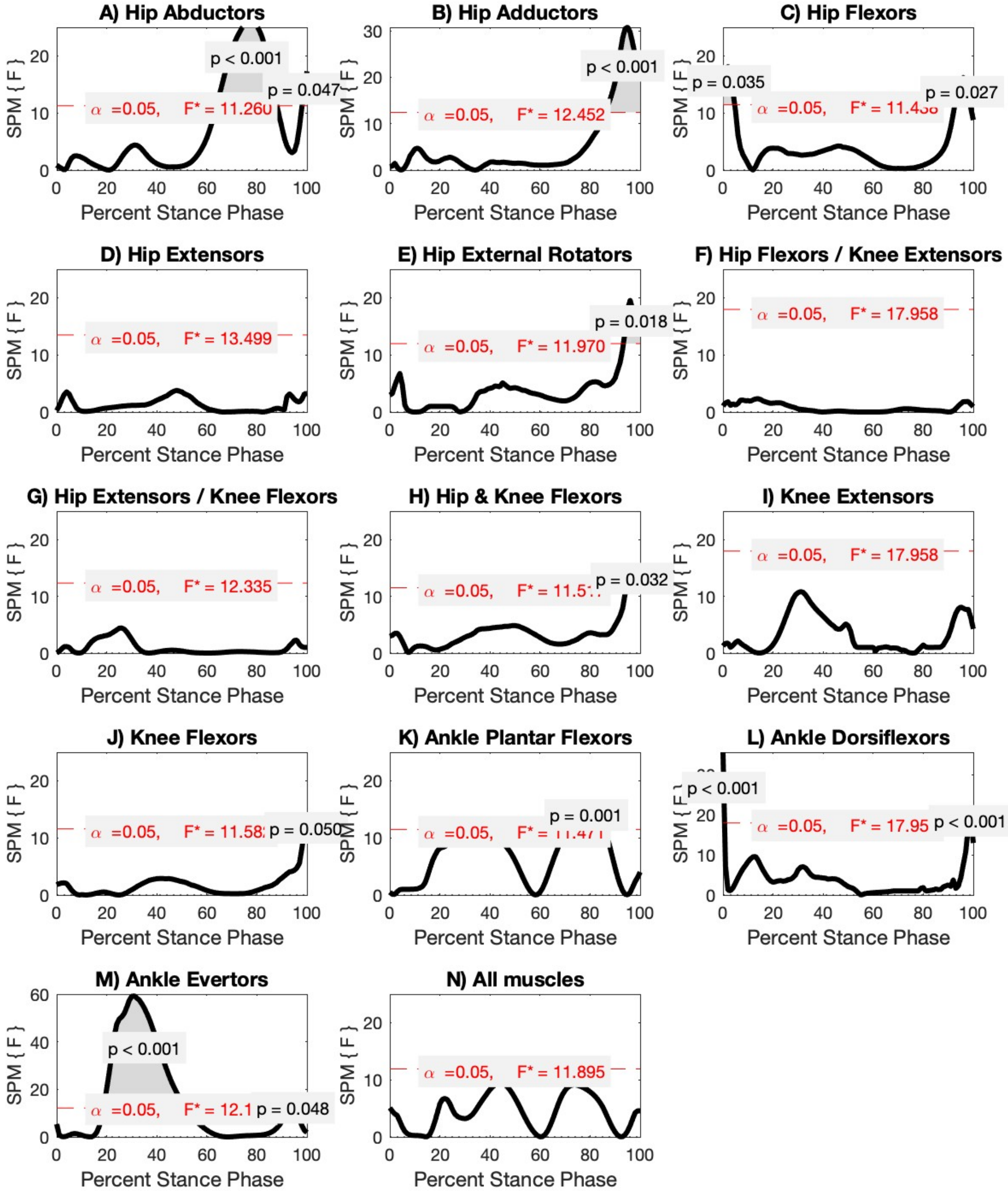

Fig. S12. Differences in normalized functional group muscle force profiles for apex step during a 90° with a sidestep revealed by the SPM. The statistically significant differences (for $p < 0.05$) are marked on the horizontal bar.

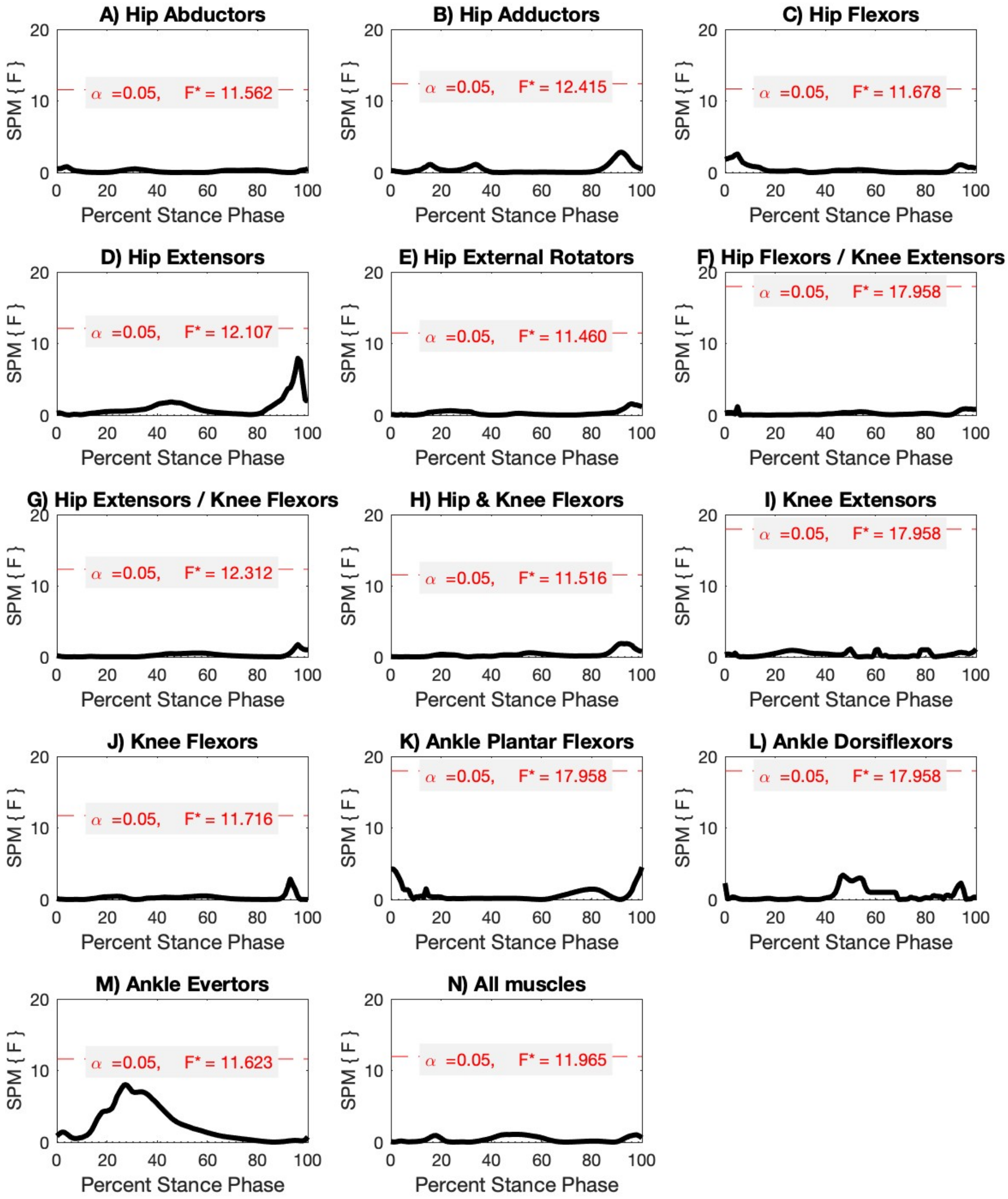

Fig. S13. Differences in normalized functional group muscle force profiles for initiation step during a 45° with a sidestep revealed by the SPM. The statistically significant differences (for $p < 0.05$) are marked on the horizontal bar.

Biology Open • Supplementary information

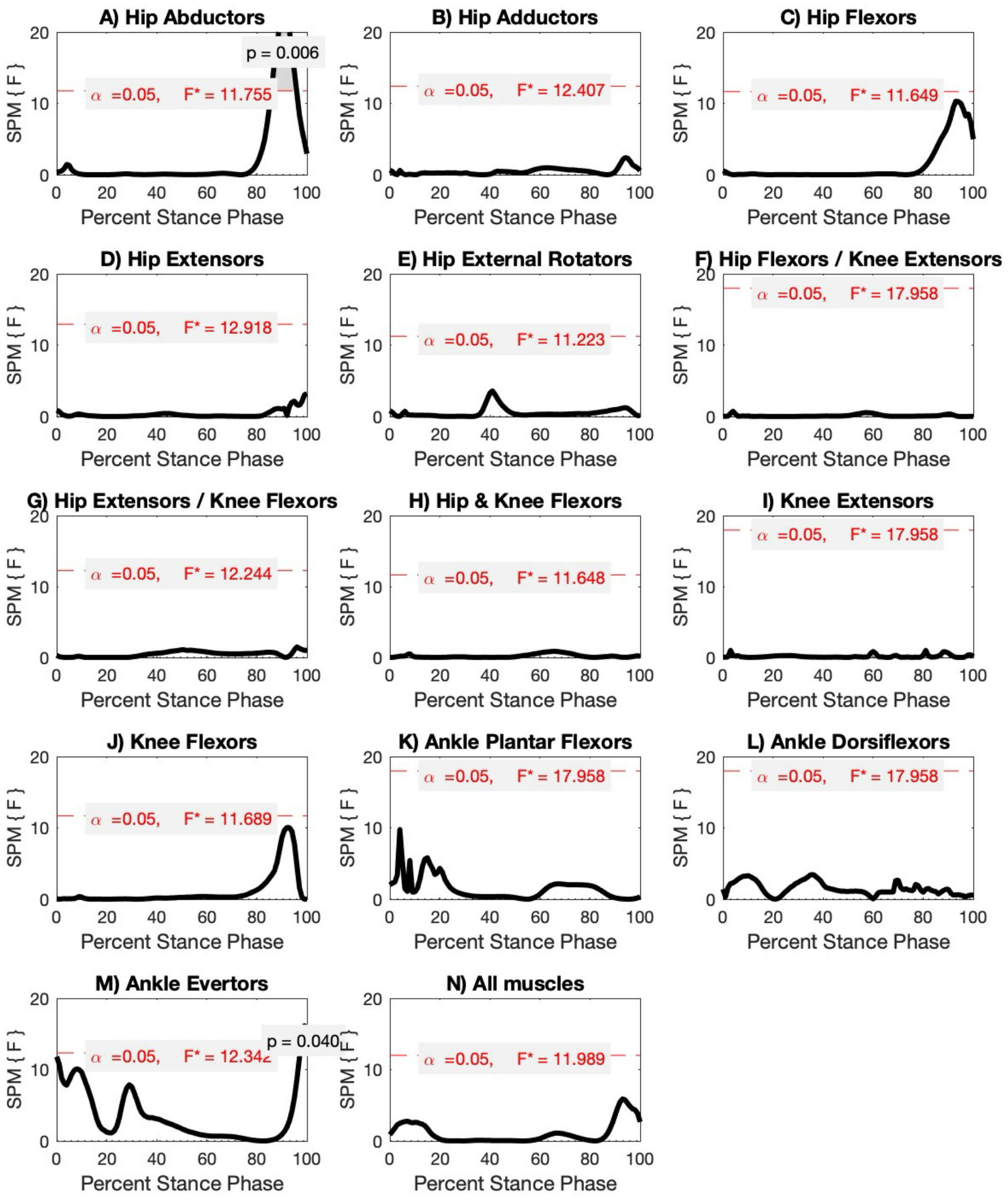

Fig. S14. Differences in normalized functional group muscle force profiles for initiation step during a 45° with a crossover revealed by the SPM. The statistically significant differences (for $p < 0.05$) are marked on the horizontal bar.

Biology Open • Supplementary information

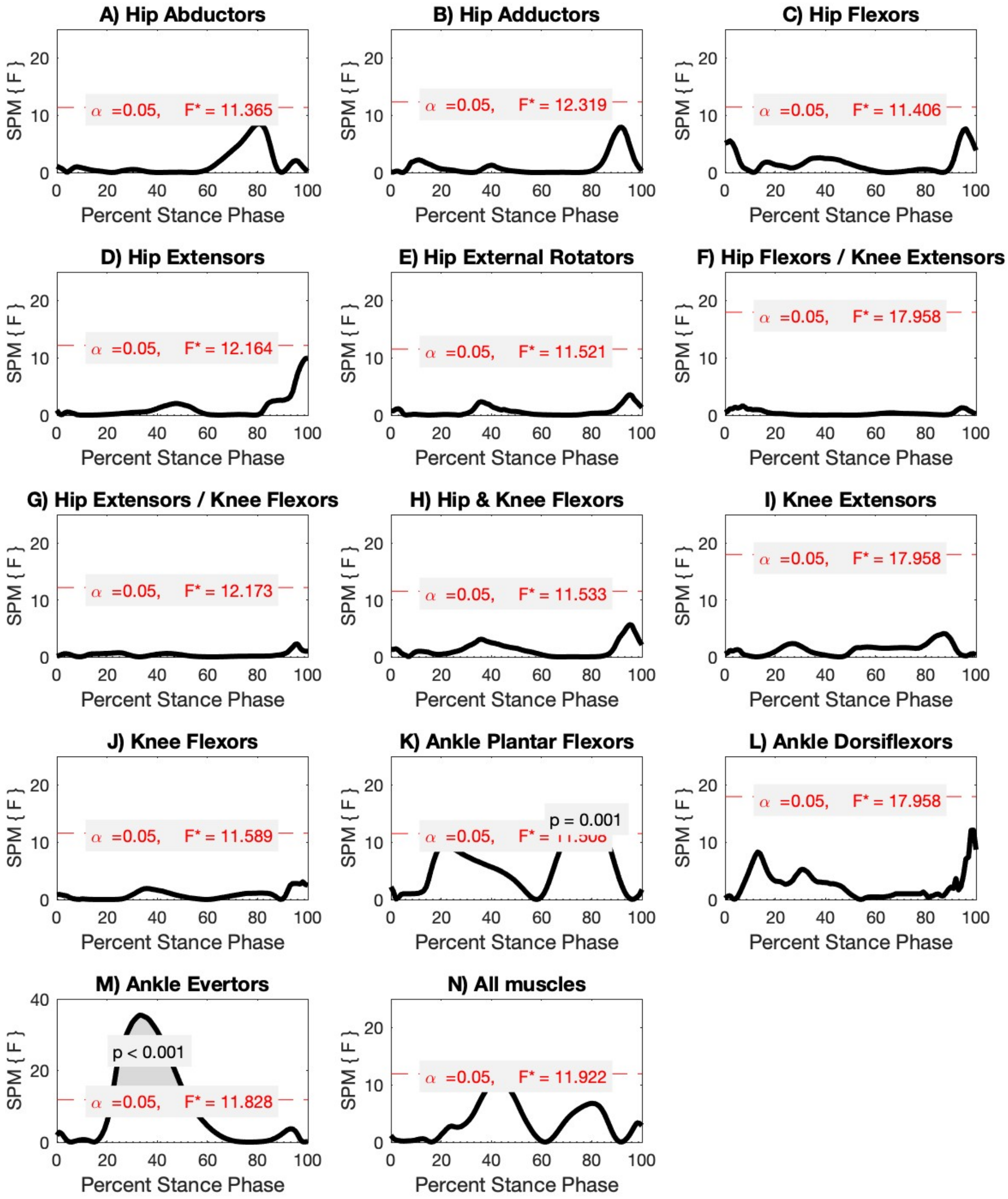

Fig. S15. Differences in normalized functional group muscle force profiles for initiation step during a 45° with a sidestep revealed by the SPM. The statistically significant differences (for $p < 0.05$) are marked on the horizontal bar.

Biology Open • Supplementary information

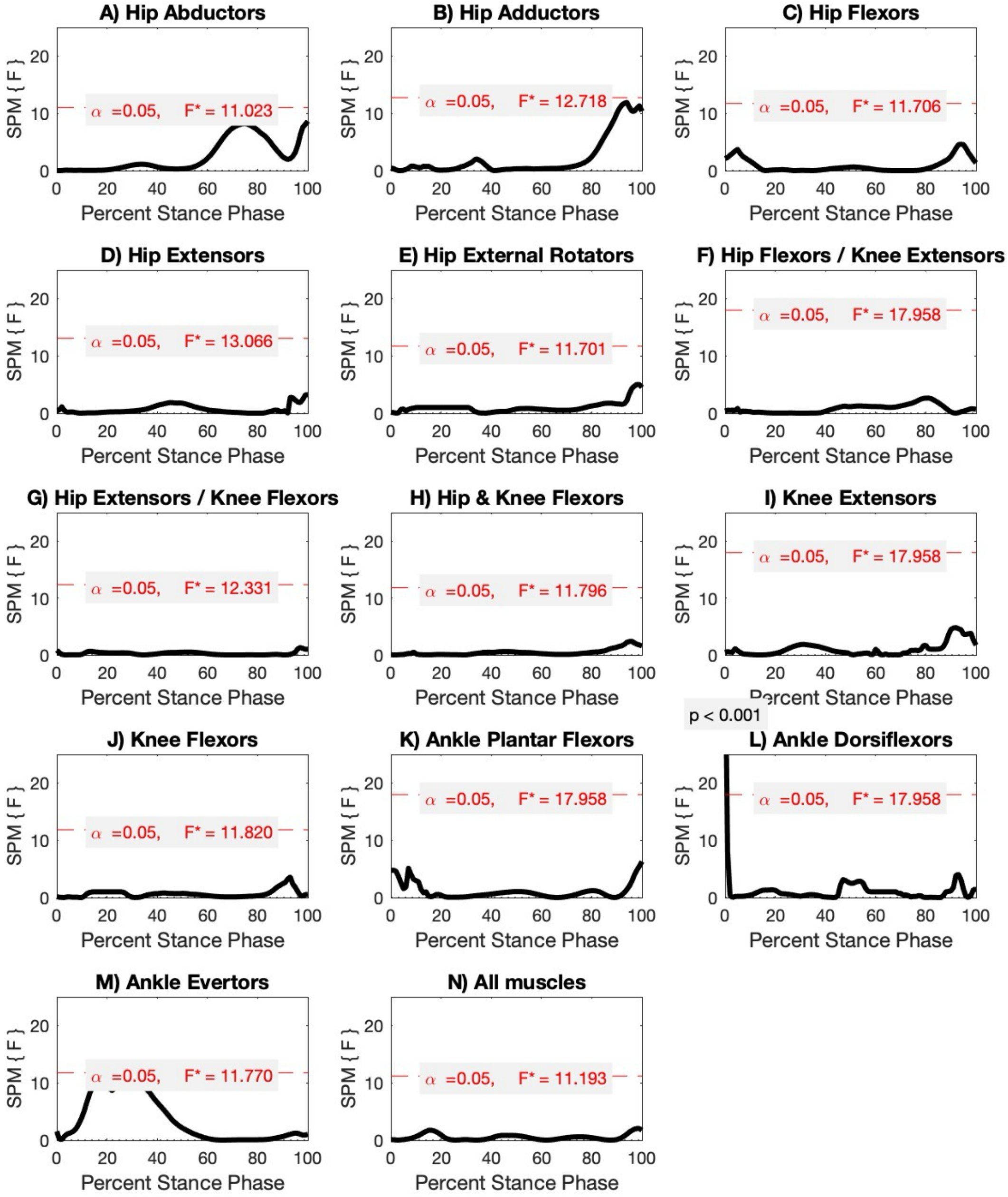

Fig. S16. Differences in normalized functional group muscle force profiles for apex step during a 45° with a sidestep revealed by the SPM. The statistically significant differences (for $p < 0.05$) are marked on the horizontal bar.

Biology Open • Supplementary information

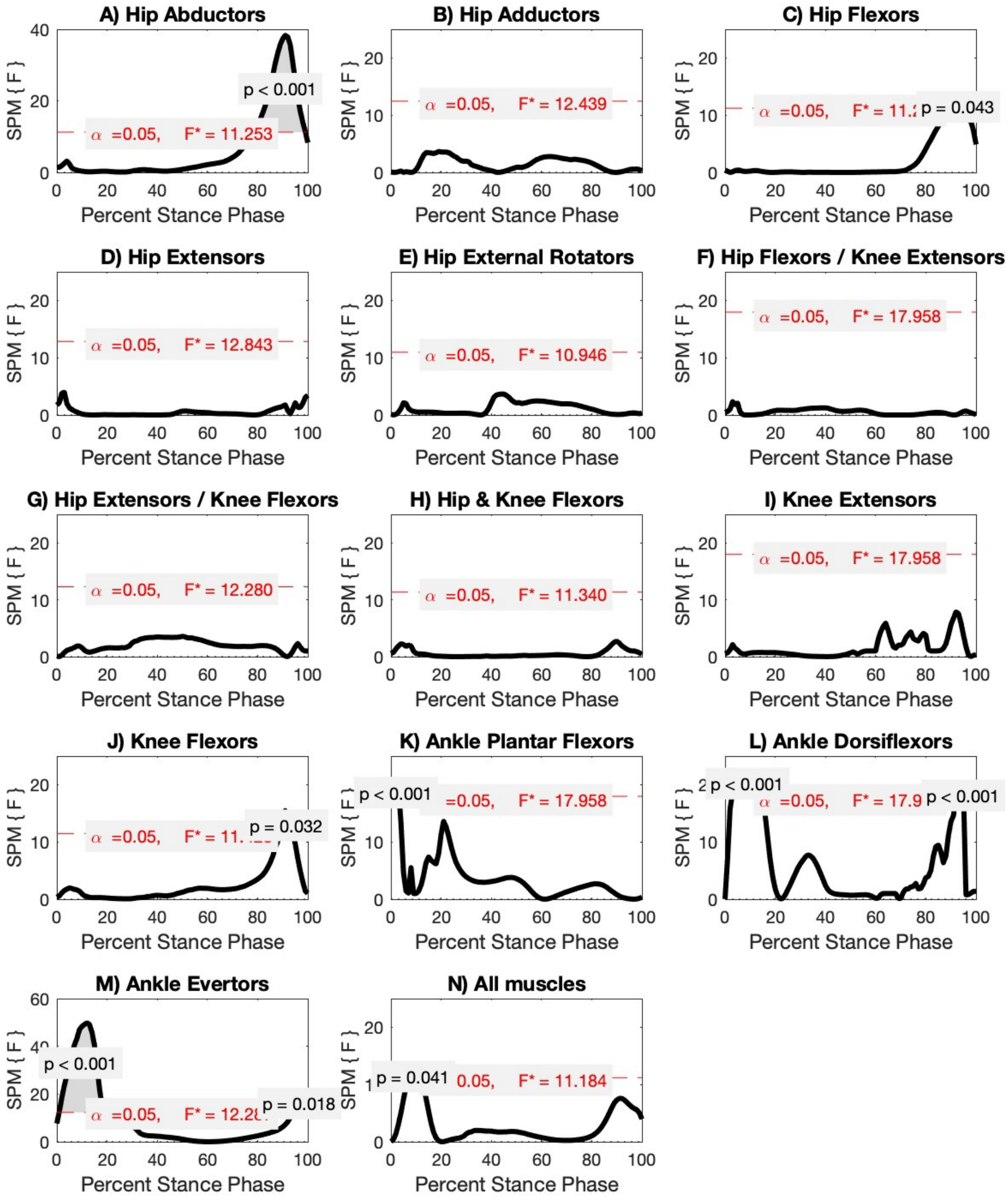

Fig. S17. Differences in normalized functional group muscle force profiles for apex step during a 45° with a crossover revealed by the SPM. The statistically significant differences (for $p < 0.05$) are marked on the horizontal bar.

Biology Open • Supplementary information

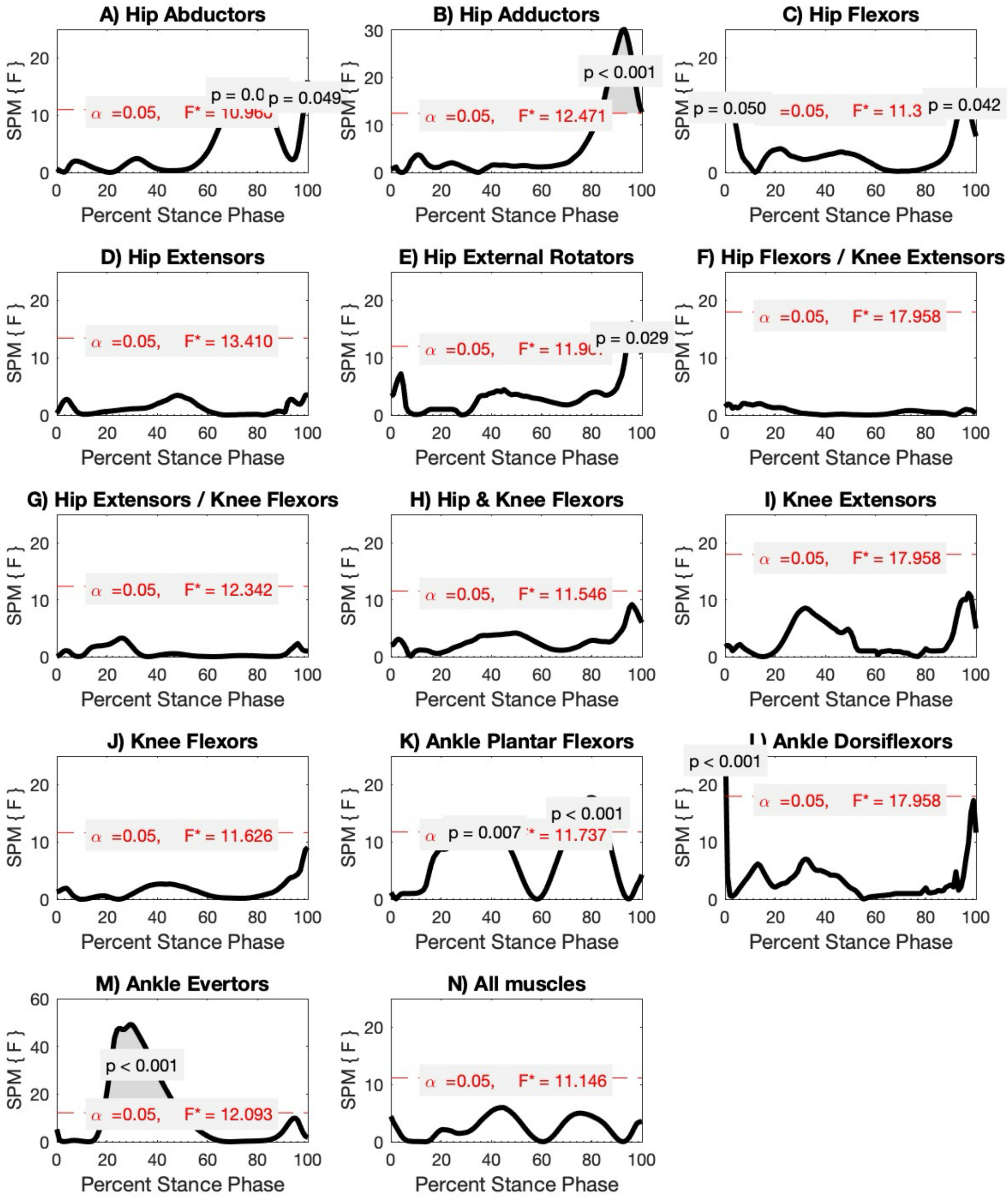

Fig. S18. Differences in normalized functional group muscle force profiles for apex step during a 90° with a sidestep revealed by the SPM. The statistically significant differences (for $p < 0.05$) are marked on the horizontal bar.

Biology Open • Supplementary information

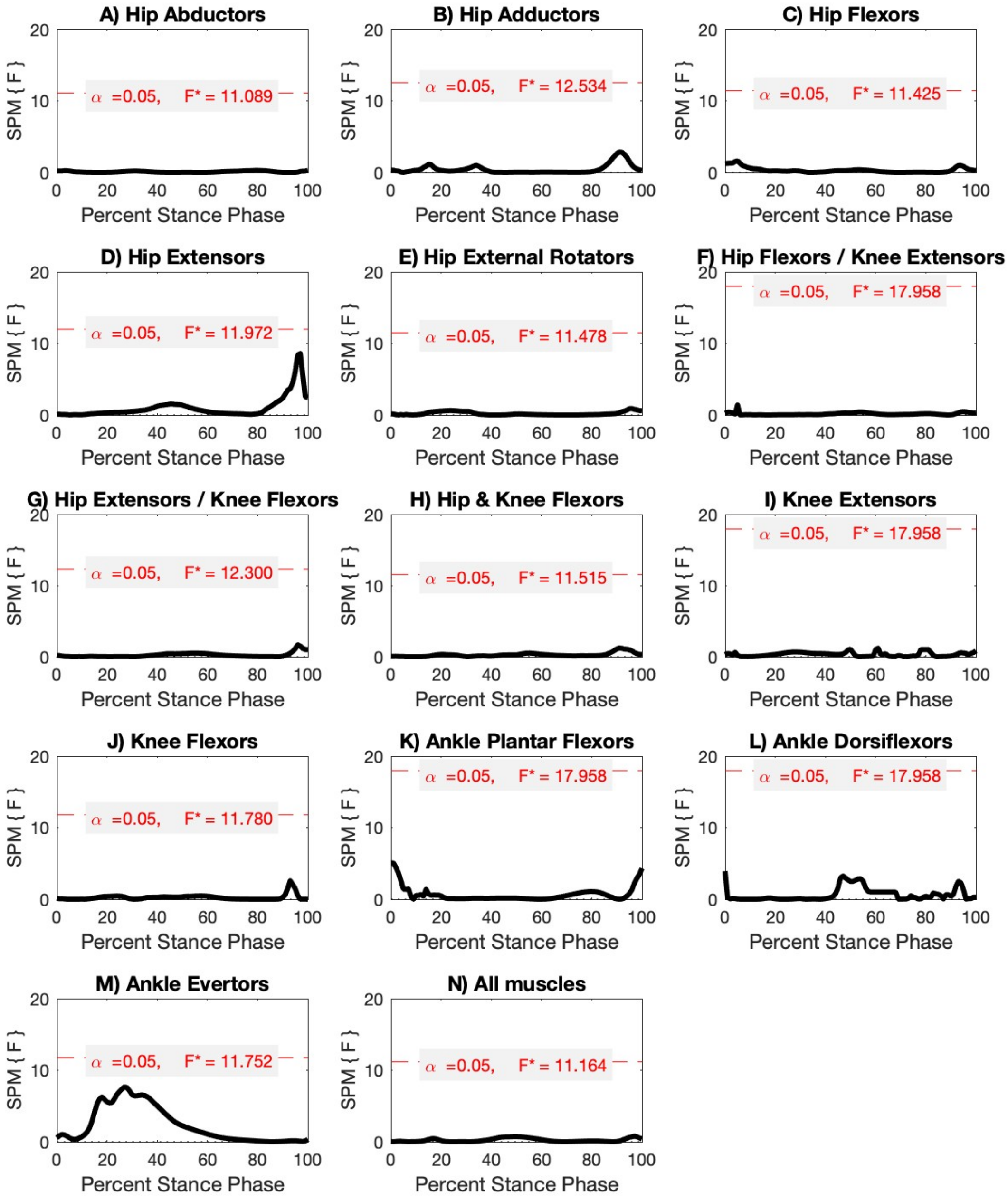

Fig. S19. Differences in normalized functional group muscle force profiles for initiation step during a 45° with a sidestep revealed by the SPM. The statistically significant differences (for $p < 0.05$) are marked on the horizontal bar.

Biology Open • Supplementary information

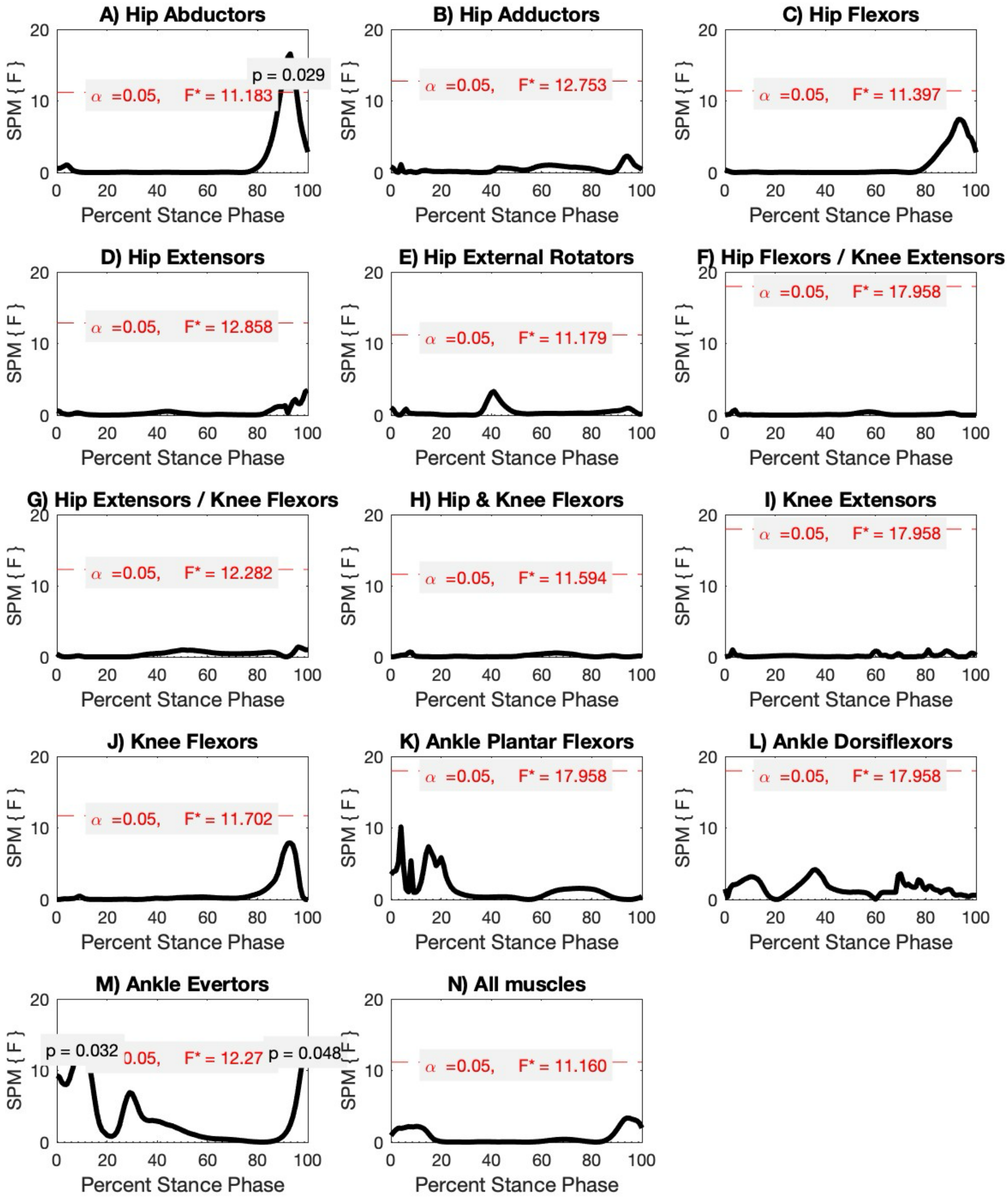

Fig. S20. Differences in normalized functional group muscle force profiles for initiation step during a 45° with a crossover revealed by the SPM. The statistically significant differences (for $p < 0.05$) are marked on the horizontal bar.

Biology Open • Supplementary information

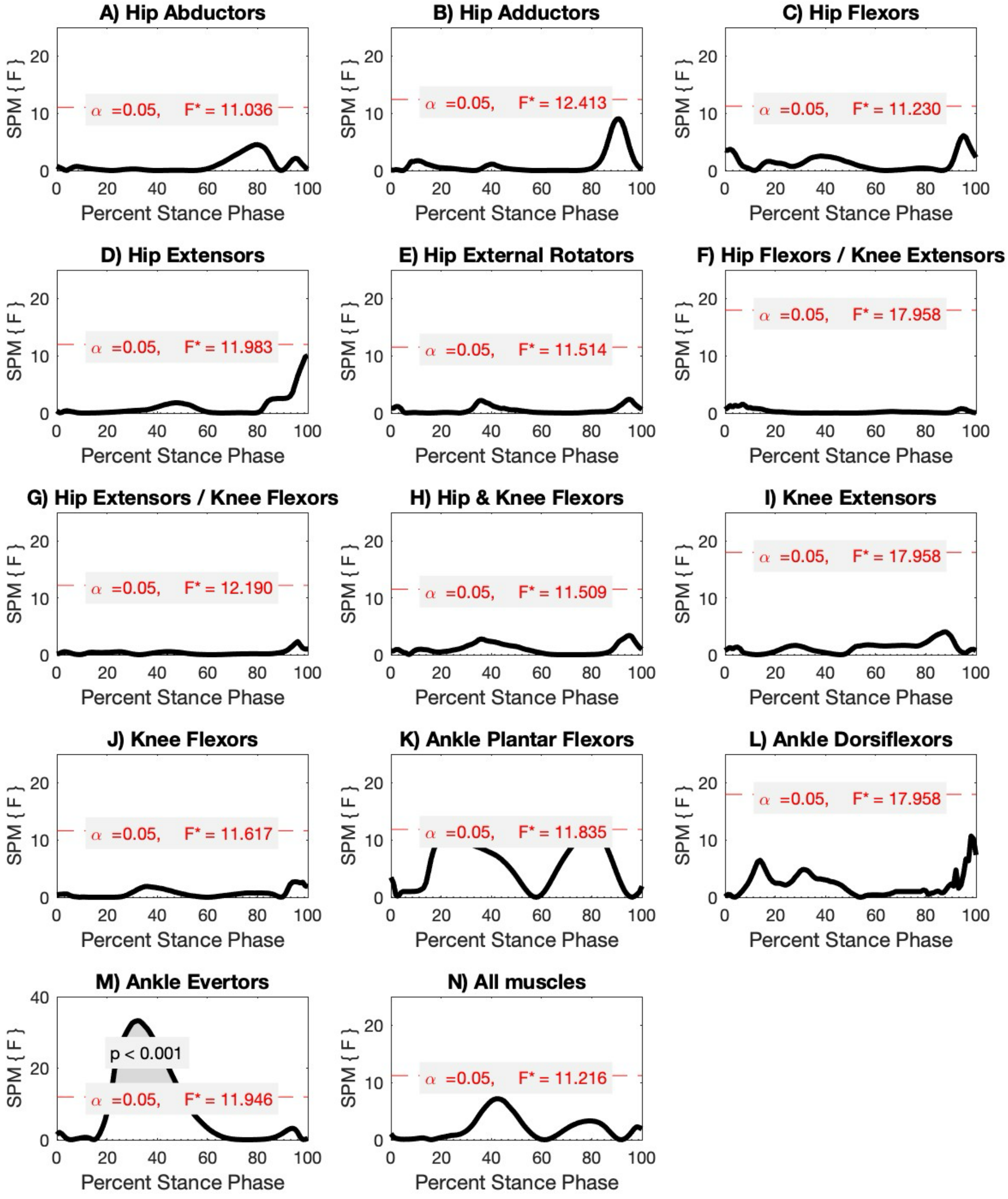

Fig. S21. Differences in normalized functional group muscle force profiles for initiation step during a 90° with a sidestep revealed by the SPM. The statistically significant differences (for $p < 0.05$) are marked on the horizontal bar.

Biology Open • Supplementary information

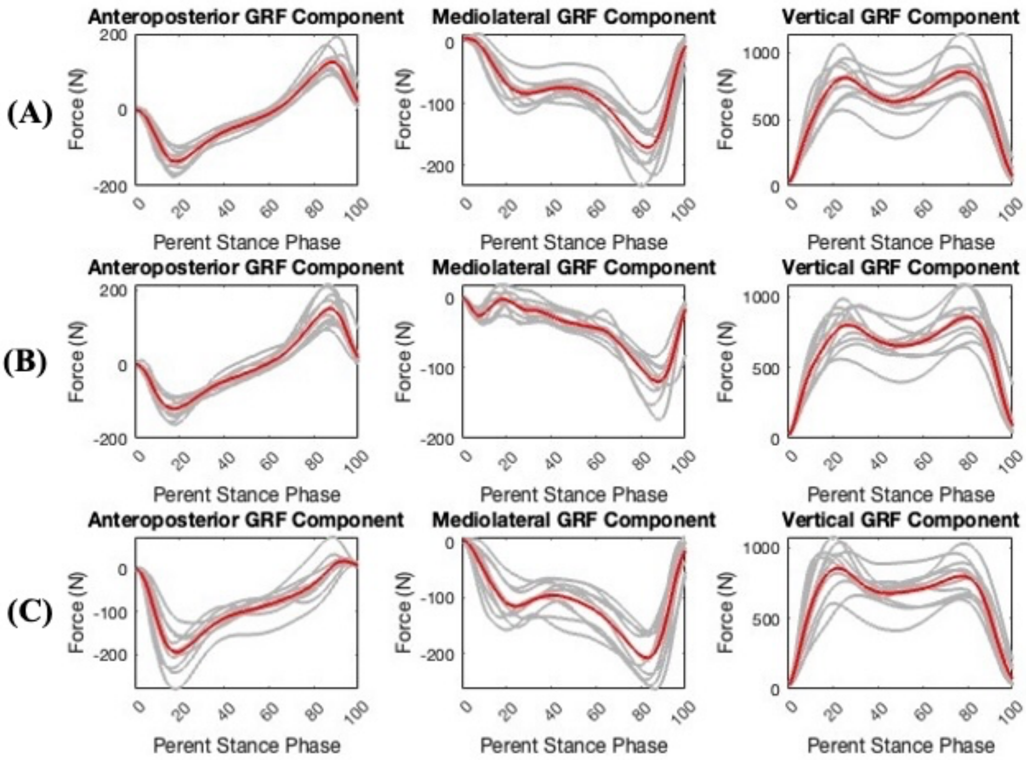

Fig. S22. Ground reaction force profiles during stance phase of the apex step during a 45° with a sidestep (A), 45° with a crossover (B), and 90° with a sidestep (C). Grey lines are average curve for each participant (two to five stance phases per participant). Red line is average of the ten participant average curves.

Biology Open • Supplementary information

Table S1. Participant anthropometrics including age (male: 31.8 ± 13.8 years; female: 34 ± 5.4 years), stature (male: 1.66 ± 0.05 m; female: 1.77 ± 0.05 m), body mass (male: 73.4 ± 17.5 kg; female: 85.2 ± 6.2 kg), BMI (male: 26.5 ± 5.5 kg/m²; female: 27.2 ± 1.6 kg/m²). and average velocity for each walking condition. Velocity was calculated using the glabella marker.

ID	Sex	Age	Mass (kg)	Stature (m)	BMI (kg/m²)	Average Velocity of straight path (m/s)	Average Velocity of 45° turn with a sidestep (m/s)	Average Velocity of 45° turn with a crossover (m/s)	Average Velocity of 90° turn with a sidestep (m/s)
1	f	29	53.1	1.59	21.0	1.34	1.23	1.34	1.24
2	m	29	88.9	1.80	27.4	1.29	1.29	1.23	1.20
3	f	21	69	1.72	23.3	1.12	1.12	1.12	1.13
4	m	42	87.3	1.72	29.5	1.42	1.33	1.35	1.30
5	f	55	82.3	1.69	28.8	1.25	1.21	1.24	1.20
6	f	32	64.1	1.62	24.4	1.15	1.11	1.09	1.10
7	m	36	74.3	1.72	25.1	1.18	1.12	1.13	1.10
8	f	22	98.5	1.68	34.9	1.19	1.15	1.16	1.15
9	m	34	89.2	1.82	26.9	1.18	1.21	1.20	1.21
10	m	29	86.5	1.79	27.0	1.24	1.18	1.19	1.15

Appendix S1: Definition of each external landmark.

Upper Back Plate – a marker plate consisting of 4 infrared markers placed in the center of the thoracic region.

- Left Superior upper back (LSUB) – upper left marker on the marker plate placed on the upper back
- Left inferior upper back (LIUS) - lower left marker on the marker plate placed on the upper back
- Right superior upper back (RSUB) - upper right marker on the marker plate placed on the upper back
- Right inferior upper back (RIUB) - lower right marker on the marker plate placed on the upper back

Lower Back Plate - a marker plate consisting of 4 infrared markers placed in the center of the lumbar region.

- Left superior lower back (LSLB) - upper left marker on the marker plate placed on the lower back
- Left inferior lower back (LILB) - lower left marker on the marker plate placed on the lower back
- Right superior lower back (RSLB) - upper right marker on the marker plate placed on the lower back
- Right inferior lower back (RILB) - lower right marker on the marker plate placed on the lower back

Thigh Plate - a marker plate consisting of 4 infrared markers placed in the center of the thigh.

- Anterior superior thigh (AST) - upper left marker on the marker plate placed on the thigh
- Anterior inferior thigh (AIT) - upper right marker on the marker plate placed on the thigh
- Posterior superior thigh (PST) - lower left marker on the marker plate placed on the thigh
- Posterior inferior thigh (PIT) - lower right marker on the marker plate placed on the thigh

Skin Markers:

- Glabella (glabella) – the location on the skin over the most prominent point of the glabella.

- Acromion process (Acrom) - the superior location on the skin over the most prominent point of the acromion process.
- Anterior superior iliac spine (ASIS) - the location on the skin over the most prominent point of the anterior superior iliac spine.
- Iliac crest (IC) – the location on the skin over the most prominent point of the iliac crest.
- Medial knee (MedKnee) – the location on the skin on the most prominent point of the tibial plateau
- Lateral knee (LatKnee) – the location on the skin on the most prominent point of the fibular head.
- Tibial tuberosity (TibTub) - the location on the skin over the most prominent point of the tibial tuberosity
- Patella (Pat) - the location on the skin over the superior most prominent point of the patella
- Lateral Malleolus (LATMAL) – the location on the skin over the most prominent point of lateral malleolus
- Medial Malleolus (MEDMAL) – the location on the skin over the most prominent point of medial malleolus
- 1st metatarsal head (MT1) – the location on the skin over the center of the head of the 1st metatarsal.
- 1st metatarsal base (MT1Base) - the location on the skin over the center of the bast of the 1st metatarsal.
- 5st metatarsal head (MT5) – the location on the skin over the center of the head of the 5st metatarsal.
- Styloid process of the fifth metatarsal (SPMT5) – the location on the skin over the most prominent point of the Styloid process of the fifth metatarsal
- Dorsal navicular (DNAV) – the location on the skin over the dorsal most prominent point of the navicular.
- Heel (Heel)– the location on the skin over the location where the Achilles tendon attaches to the calcaneus.
- Navicular tubercle (NAVTUB) – the location on the skin over the most prominent point of the navicular tubercle

Biology Open • Supplementary information

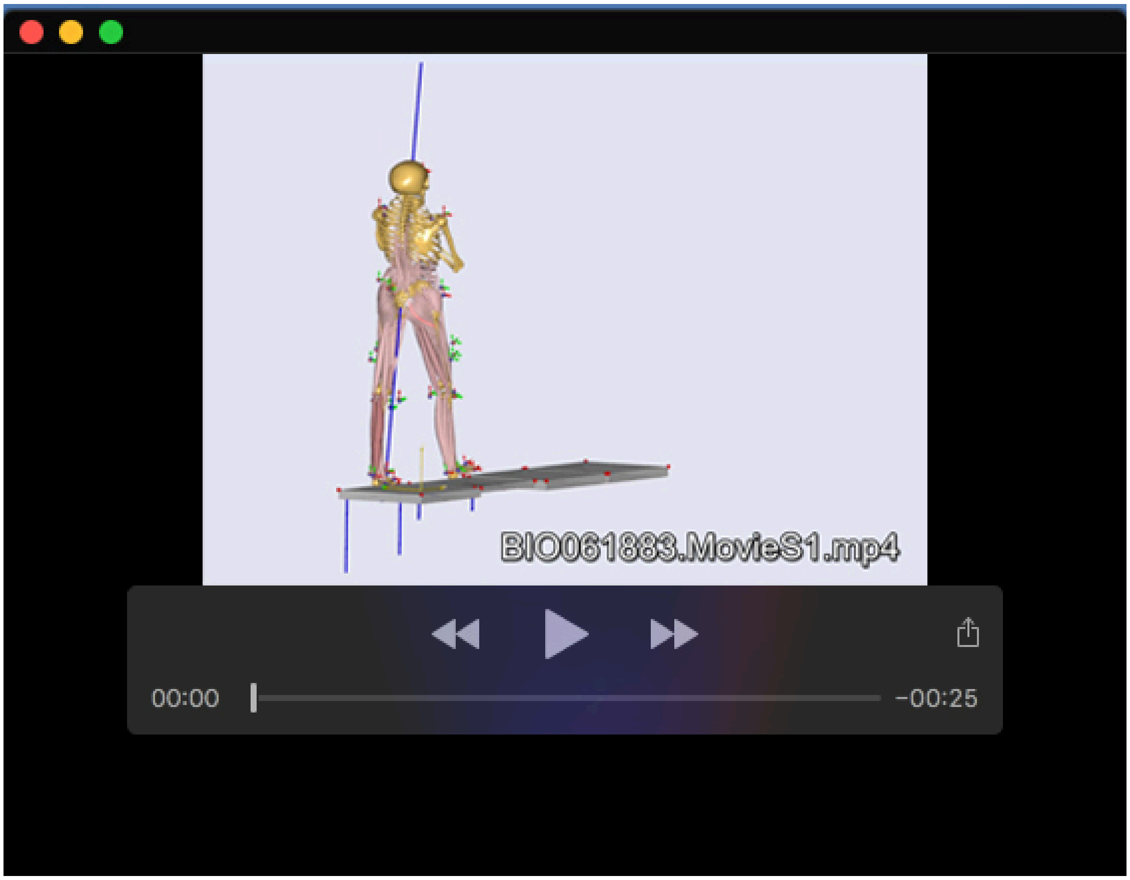

Movie 1. MoCap video for the straight path walking conditions. The video shows the model (represented by skeletal and muscular elements), four force plates (grey), the ground reaction force vector (blue line through model), force plate readings (blue lines below the force plate), experimental marker locations (blue spheres), virtual markers (red spheres with coordinate system arrows) and global coordinate system (yellow arrows). Participant's arms swung naturally while walking and were not crossed over their chest during the trial.

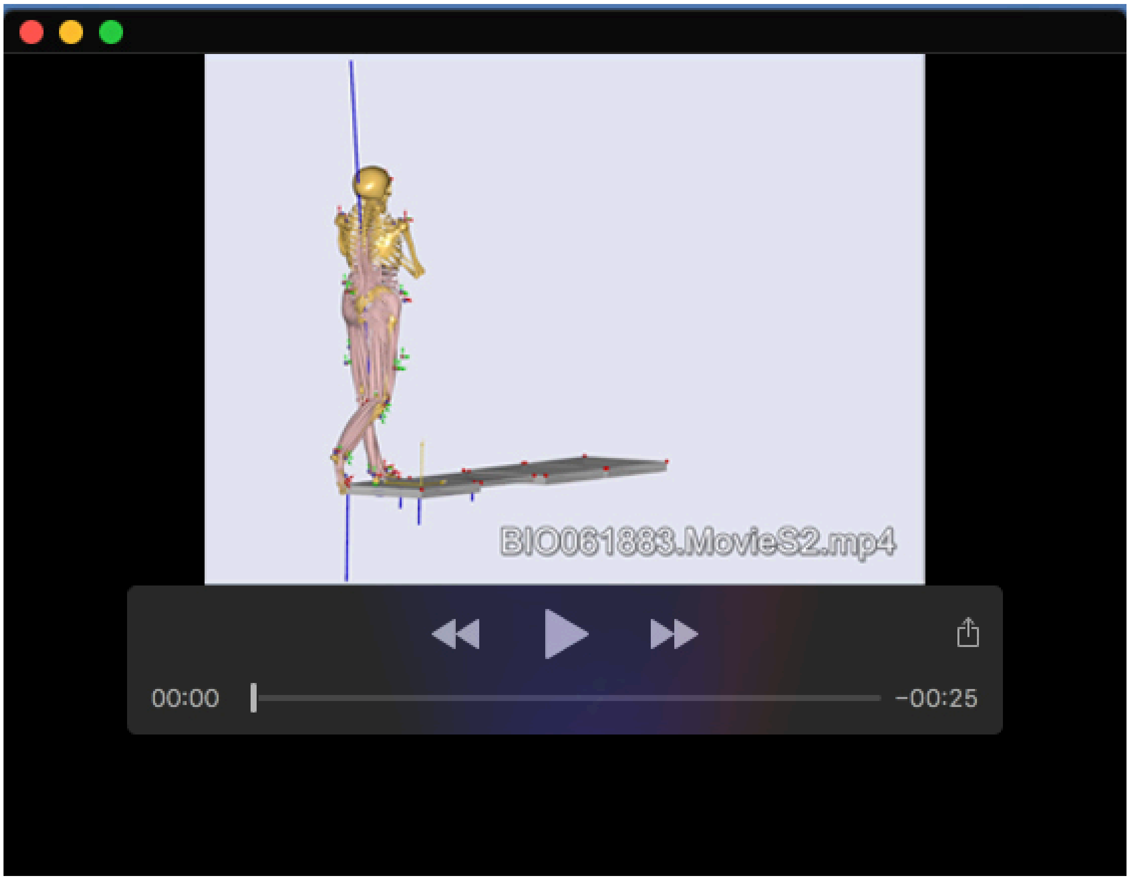

Movie 2. MoCap video for the 45° with a side-step condition. The video shows the model (represented by skeletal and muscular elements), four force plates (grey), the ground reaction force vector (blue line through model), force plate readings (blue lines below the force plate), experimental marker locations (blue spheres), virtual markers (red spheres with coordinate system arrows) and global coordinate system (yellow arrows). Participant's arms swung naturally while walking and were not crossed over their chest during the trial.

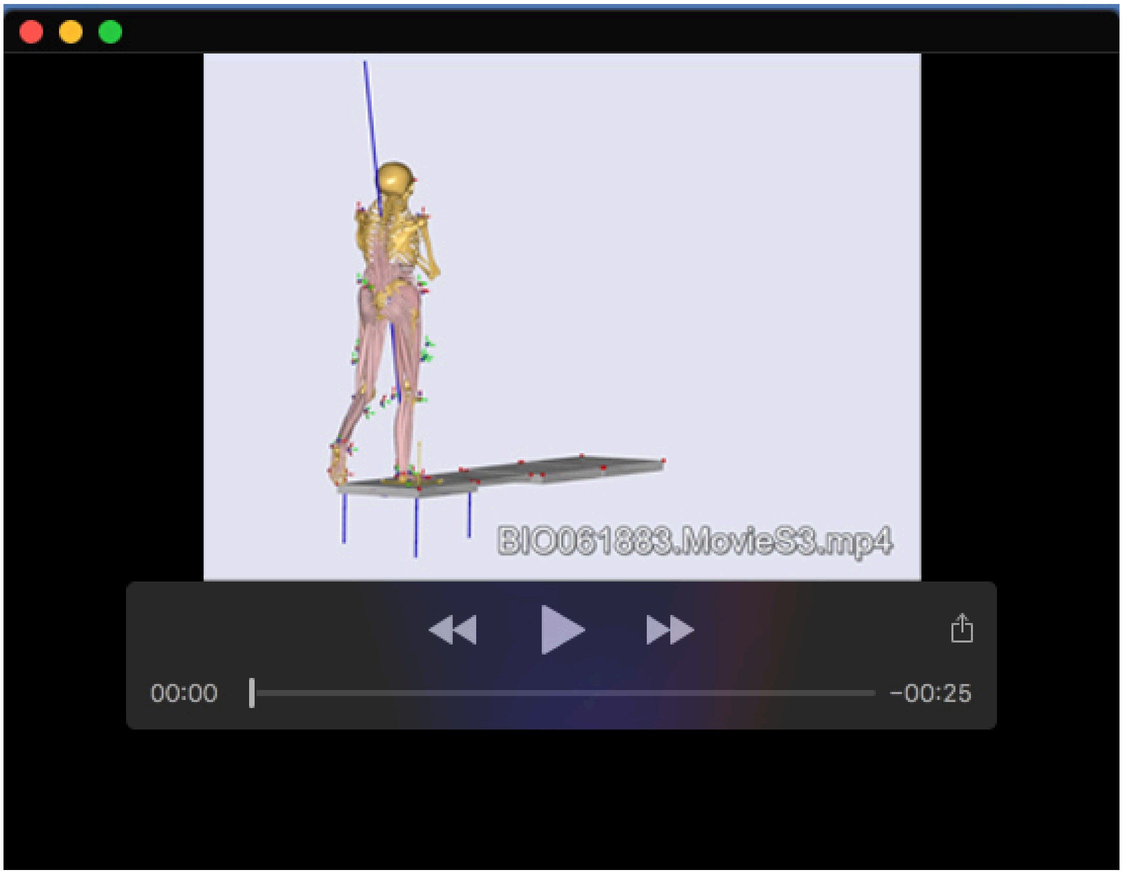

Movie 3. MoCap video for the 45° with a cross over condition. The video shows the model (represented by skeletal and muscular elements), four force plates (grey), the ground reaction force vector (blue line through model), force plate readings (blue lines below the force plate), experimental marker locations (blue spheres), virtual markers (red spheres with coordinate system arrows) and global coordinate system (yellow arrows). Participant's arms swung naturally while walking and were not crossed over their chest during the trial.

Biology Open • Supplementary information

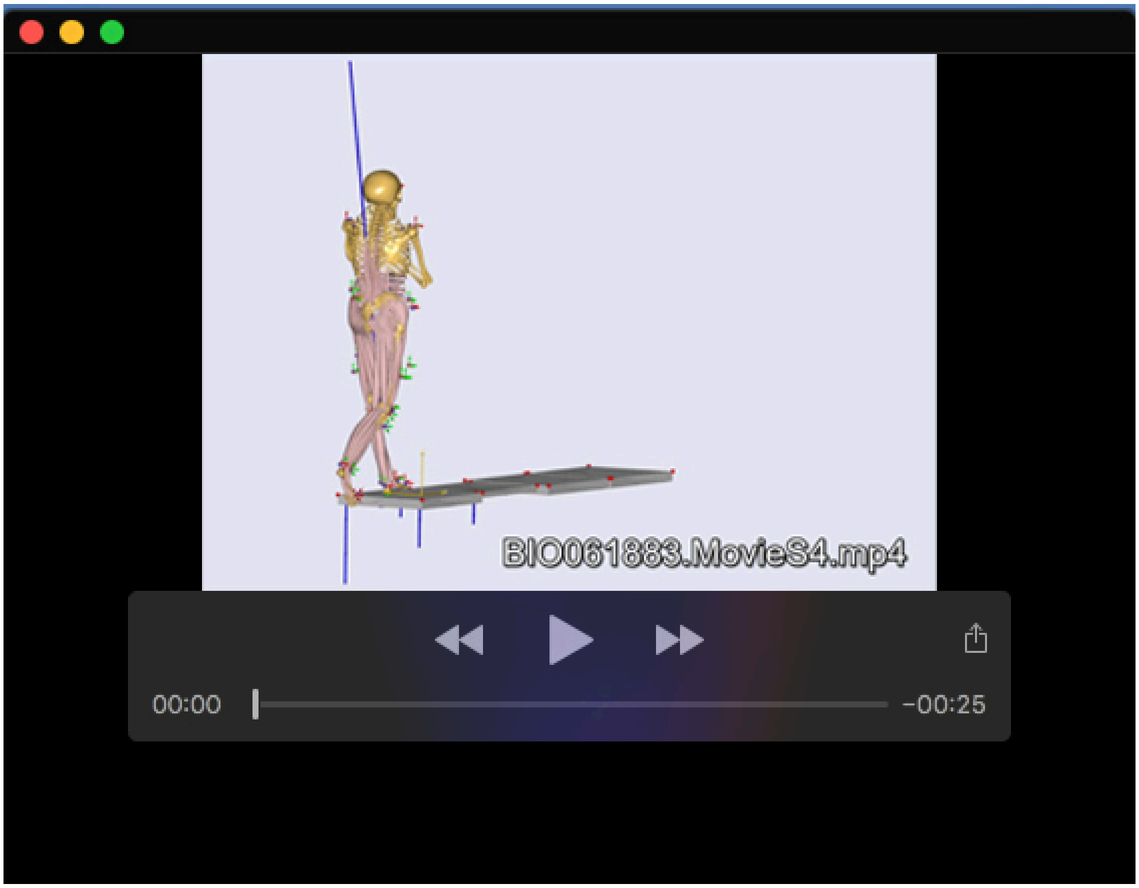

Movie 4. MoCap video for the 90° with a side-step condition. The video shows the model (represented by skeletal and muscular elements), four force plates (grey), the ground reaction force vector (blue line through model), force plate readings (blue lines below the force plate), experimental marker locations (blue spheres), virtual markers (red spheres with coordinate system arrows) and global coordinate system (yellow arrows). Participant's arms swung naturally while walking and were not crossed over their chest during the trial.

References

Sylvester, A.D., Lautzenheiser, S.G., Kramer, P.A., 2021. Muscle forces and the demands of human walking. Biol Open 10. https://doi.org/10.1242/BIO.058595
